# Lipidomic Profiling of PFOA-Exposed Mouse Liver by Multi-Modal Mass Spectrometry Analysis

**DOI:** 10.1021/acs.analchem.2c05470

**Published:** 2023-04-07

**Authors:** Charlotte B. A. Stoffels, Tina B. Angerer, Hervé Robert, Nathalie Poupin, Laila Lakhal, Gilles Frache, Muriel Mercier-Bonin, Jean-Nicolas Audinot

**Affiliations:** † Department of Materials Research and Technology, Luxembourg Institute of Science and Technology, Belvaux 4422, Luxembourg; ‡ Faculty of Science, Technology and Medicine, University of Luxembourg, Esch-sur-Alzette 4365, Luxembourg; § Toxalim, Université de Toulouse, INRAE, INP-ENVT, INP-EI-Purpan, Université de Toulouse 3 Paul Sabatier, Toulouse 31027, France

## Abstract

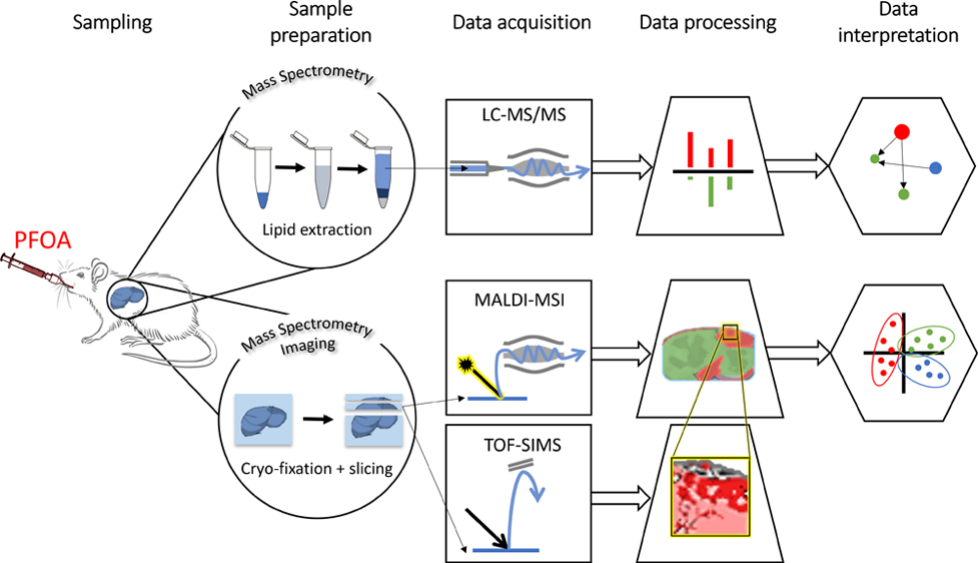

Perfluorooctanoic acid (PFOA) is a synthetic perfluorinated chemical classified as a persistent organic pollutant. PFOA has been linked to many toxic effects, including liver injury. Many studies report that PFOA exposure alters serum and hepatic lipid metabolism. However, lipidomic pathways altered by PFOA exposure are largely unknown and only a few lipid classes, mostly triacylglycerol (TG), are usually considered in lipid analysis. Here, we performed a global lipidomic analysis on the liver of PFOA-exposed (high-dose and short-duration) and control mice by combining three mass spectrometry (MS) techniques: liquid chromatography with tandem mass spectrometry (LC–MS/MS), matrix-assisted laser desorption ionization mass spectrometry imaging (MALDI-MSI), and time-of-flight secondary ion mass spectrometry (TOF-SIMS). Among all hepatic lipids identified by LC–MS/MS analysis, more than 350 were statistically impacted (increased or decreased levels) after PFOA exposure, as confirmed by multi-variate data analysis. The levels of many lipid species from different lipid classes, most notably phosphatidylethanolamine (PE), phosphatidylcholine (PC), and TG, were significantly altered. Subsequent lipidomic analysis highlights the pathways significantly impacted by PFOA exposure, with the glycerophospholipid metabolism being the most impacted, and the changes in the lipidome network, which connects all the lipid species together. MALDI-MSI displays the heterogeneous distribution of the affected lipids and PFOA, revealing different areas of lipid expression linked to PFOA localization. TOF-SIMS localizes PFOA at the cellular level, supporting MALDI-MSI results. This multi-modal MS analysis unveils the lipidomic impact of PFOA in the mouse liver after high-dose and short-term exposure and opens new opportunities in toxicology.

Since the 1950s, perfluoroalkylated substances (PFAS), such as perfluorooctanoic acid (PFOA), have been widely used in many industrial applications, including carpeting, firefighting foam, and textiles,^1−4^ and are therefore ubiquitous in our surroundings. However, these chemicals are toxic and persistent. They accumulate in living organisms, including humans,^5^ as well as in a variety of wildlife,^6^ leading to major health issues.^52^ PFOA has been detected in human body fluids and tissues and related to hepatotoxicity, genotoxicity, and reproductive toxicity.^7^ At the European level, it has been identified as a substance of high concern by the European Chemicals Agency under the REACH regulation (EC 2017), and in 2019, it was listed together with its salts and PFOA-related compounds in Annex A (elimination) of the Stockholm Convention.

In rodents, liver toxicity is one of the most common reported effects of PFAS.^8^ Many studies have linked PFAS exposure with fatty liver disease (hepatic steatosis), the most common liver disease in humans, which is characterized by excessive triacylglycerol (TG) accumulation within hepatocytes. Fatty liver disease can progress to an inflammatory state (steatohepatitis), and at worst, to cirrhosis and hepatocellular carcinoma.^9^ In particular, PFOA exposure has led to extensive micro- and macro-vesicular steatosis in hepatocytes and the accumulation of TG in the liver.^10−13^ Other studies have shown that PFOA exposure in mice and rats resulted in weight loss, liver injury, liver enlargement, and disorders of lipid metabolism.^14,15^ PFOA-induced hepatocellular lipid dysmetabolism has been validated both in vivo (human and animal studies) and in vitro.^16,17^ However, some controversies exist between human and animal data regarding the level change of some serum and intrahepatic lipids due to exposure conditions (doses and duration) and intrinsic differences.^18^ Structurally similar to fatty acids, PFAS, including PFOA, have been found to bind to peroxisome proliferator-activated receptors (PPARs) and particularly to PPAR-α, which is a key transcription factor in lipid metabolism.^19−21^

Even though the disturbance of lipid homeostasis associated with PFAS exposure has been extensively reported, the underlying mechanisms remain largely unknown. The majority of the hepatological studies available on PFOA focus on histological analysis [light and electron microscopy (LM and EM, respectively)] to investigate lipid content and cellular morphology using staining agents (e.g., Oil Red O or Hematoxylin & Eosin (H&E) in LM and osmium tetroxide in EM), on TG assays and on gene expression profiling.^19−21^ Few studies performed lipid analysis using analytical methods^22^ and most focus on small lipids such as fatty acids^11^ and cholesterol.^23,24^ Moreover, there are no data on the hepatocellular localization of PFOA, which could strengthen our understanding of molecular mechanisms underlying PFOA exposure.

Mass spectrometry (MS) techniques, including liquid chromatography with tandem MS (LC−MS/MS) and MS imaging (MSI), are well suited for identifying and localizing molecules such as lipids and toxicants (e.g., PFOA) in biological samples.^25,26^ MSI techniques scan the sample surface and record one mass spectrum at each location, providing the distribution of all detected species. Common MSI techniques are time-of-flight secondary ion MS (TOF-SIMS) and matrix-assisted laser desorption ionization (MALDI)-MSI. In TOF-SIMS analysis, the sample surface is scanned by an ion beam and the technique is used for the localization of elements, small molecules, and molecular fragments with a submicron lateral resolution.^27^ MALDI-MSI is a technique in which the sample surface is covered with a matrix and subsequently scanned by a laser beam, allowing the localization of numerous intact molecules, such as lipids, typically with a 10-μm lateral resolution^28^ although higher resolution analyses have been demonstrated.^29^

MS-based lipidomics, i.e., the large-scale study of lipids using MS methods and the biological interpretation of these data, is gaining importance in toxicology due to major analytical technology advances in recent years.^30,31^ Several thousands of lipids exist and interact via many pathways and networks. Evaluating and understanding changes in these networks in response to cellular environment alterations, in association with the development of a disease, are crucial to deciphering cell metabolism and related molecular mechanisms.^32,33^

To the best of our knowledge, the hepatocellular localization of PFOA and global mouse liver lipidomic analysis after exposure have never before been investigated. Hence, in our current study, multi-modal MS analyses, including LC−MS/MS, TOF-SIMS, and MALDI-MSI, were used to unravel the fate and lipidomic impacts of PFOA in the mouse liver after a 3-day acute exposure at high dose (100 mg/kg bw/day).

## Experimental Section

### Chemicals

Chemicals and solvents (analytical grade) were purchased from the following sources: gelatin (Sigma-Aldrich, USA), carboxymethylcellulose (CMC) (Sigma-Aldrich, USA), 1,5-diaminonaphthalene 97% (DAN) (Sigma-Aldrich, Germany), acetonitrile (ACN) (Honeywell, Germany), chloroform (Acros Organics, Belgium), and methanol (Carl Roth, Germany). All chemicals were stored, handled, and disposed of according to good laboratory practices (GLP).

### In Vivo Experiment

In this study, we used 8-week-old male C57BL/6NRj mice (Janvier Labs, France). They were kept in an animal facility under specific pathogen free (SPF) conditions at a constant temperature (21 ± 2 °C) with a day/night cycle of 12 h and had free access to water and food (Harlan Teklad 2018, Envigo, USA). All mice were acclimated to standard housing for 7 days. The experimental procedures and protocols were approved by the local ethics committee of Toulouse Midi-Pyrénées (APAFIS#21271) in accordance with European directive 2010/63/EU.

For the study, six mice were divided into two groups (three animals/group), receiving tap water (control vehicle) or PFOA (prepared in tap water) daily by oral gavage at a dose of 100 mg/kg body weight (bw)/day for 3 days. The health status of the animals was monitored daily, and no change in mouse weight was observed. After 3 days of exposure, mice were sacrificed by cervical dislocation. The liver weight was doubled after acute exposure compared to control animals, with less red steatosis-like color. Liver samples were then collected for downstream analysis with one piece for LC–MS/MS and one piece for MSI per animal.

For LC–MS/MS analysis, samples were frozen in liquid nitrogen and then stored at −80 °C, whereas samples for MSI analysis were embedded in a warm (37 °C) aliquot of gelatin (10% w/v) and CMC (2.5% w/v), frozen in isopentane previously cooled down to −160 °C in liquid nitrogen, and then stored at −80 °C.

### LC–MS/MS: Sample Preparation

Liver samples from PFOA-exposed and control mice (three animals per group) were analyzed. Mouse livers were weighted, transferred in plastic tubes, and homogenized in cold methanol (4 mL/g tissue) and deionized water (0.85 mL/g tissue) by bead beating in the Mixer Mill MM 400 (Retsch, Germany) with 0.1-mm diameter zirconium beads at 30 Hz for 3 min at 4 °C. The resulting lysates were vortexed, transferred into glass vials, submerged with cold dichloromethane (2 mL/g tissue) and deionized water (2 mL/g tissue), and vortexed again. 10 μL EquiSPLASH Internal Standard (Avanti Polar lipids, USA) was added in each sample. The vials were vortexed, kept on ice for 15 min, and then centrifuged at 3000 × g for 15 min at 4 °C, resulting in two phases separated by proteins and cellular debris. The lower dichloromethane phases (with lipophilic compounds) were carefully transferred using a Pasteur pipette into glass LC vials. The solvent was removed using the Refrigerated CentriVap Vacuum Concentrator (Labconco, USA) at 4 °C, and the samples were resuspended in 200 μL methanol and stored at −80 °C until LC–MS/MS analysis. That lipid extraction procedure was adapted from Beckonert and colleagues.^34^

### LC–MS/MS: Data Acquisition

LC–MS/MS analysis is performed using a Thermo Ultimate 3000 HPLC system coupled with a Thermo linear trap quadrupole (LTQ)/orbitrap elite high-resolution mass spectrometer (Thermo-Fisher Scientific, USA), with the sample rack and the column at 40 °C. Mobile phases used for the separation are **A:** ACN/water (40:60) containing 10 mM ammonium acetate and **B:** ACN/isopropanol (10:90) containing 10 mM ammonium acetate). HPLC was performed with a gradient elution over 40 min, 0–30 min: **A** 70% **B** 30% → **A** 0.5% **B** 99.5%, 30–40 min: **B** 99.5%, flowrate: 0.4 mL/min, injection volume: 5 μL, column: Kinetex C18 (250 × 4.6 mm^2^, 5 μm). TOP 3 DDA LC–MS/MS was performed using a 3 Da isolation window, with HCD45 fragmentation in separate runs, and full-scans over *m/z* 350–1600, in positive and negative ion mode (four runs per sample in total).

### LC–MS/MS: Data Processing and Interpretation

LC–MS/MS data were analyzed using MS–Data Independent AnaLysis (MS-dial) software for lipid annotation.^35^ Peak assignation was performed by examining tandem mass spectra for diagnostic ion fragments along with associated chain fragment information. Moreover, the isotopic profile, ion chromatogram, mass error of measured precursor, and fragment ions were examined to support identification. Data were also analyzed using Metabolomic Pathway Analysis (MetaboAnalyst, https://www.metaboanalyst.ca/) platform for pathway enrichment analysis^36,37^ and Lipid Network Explorer (LINEX, https://exbio.wzw.tum.de/linex/) platform for lipid network analysis.^38,39^ Multi-variate analyses, namely principal component analysis (PCA) and partial least squares discriminant analysis (PLS-DA), were performed using MetaboAnalyst on identified species. Data were normalized row-wise (normalization to constant sum) and column-wise (Pareto Scaling). Data were analyzed by one-way ANOVA using Graphpad Prism software (GraphPad Software Inc., USA). All results are presented as mean ± s.e.m. Differences between groups were considered statistically significant when the *p*-value < 0.05.

### MSI: Sample Preparation

Liver samples from PFOA-exposed and control mice (two animals per group) were analyzed considering two sections per sample. Cryo-fixed livers were cut into 10-μm sections and deposited on silicon wafer (Siltronix, France). Sections were kept at −80 °C. Prior to analysis, they were dried in a vacuum desiccator for 30 min. Optical images of the tissue sections were taken using an Olympus BX51 microscope (Olympus, Belgium). For MALDI analysis, sections were coated with a DAN matrix (10 mg/mL in 70% v/v ACN) using a HTX TM sprayer (HTX Technologies LLC, USA). The following parameters were applied: temperature 30 °C, flow rate 0.12 mL/min, velocity 1200 mm/min, drying time 2 s, line spacing 2.5 mm, pressure 10 psi, nozzle height 40 mm, and eight layers.

### TOF-SIMS: Data Acquisition

The TOF-SIMS analysis was performed on a TOF-SIMS V (ION-TOF GmbH, Germany) instrument. Mass spectra acquisition and imaging experiments, presented hereafter, were carried out with a 25 keV pulsed Bi_3_^+^ cluster ion source, delivering a 0.14 pA target current. The specific “burst alignment delay extraction” (BADE) mode, with a dose of 1 × 10^12^ Bi_3_^+^ ions/cm^2^, provides a good compromise between lateral resolution (submicron) and mass resolution (5000 at *m/z* 281).^40^ The analyzed area was 500 × 500 μm^2^ with a raster size of 1024 by 1024 pixels. Data were obtained in both negative and positive modes, and the secondary ion mass spectra were calibrated using C_*n*_^–^ carbon clusters and C_*x*_H_*y*_^+^ species, respectively.

### MALDI-MSI: Data Acquisition

MALDI analysis on liver sections was performed as previously described^41^ using an atmospheric pressure (AP)-MALDI ultra-high-resolution (UHR) ion source (Masstech Inc., USA) coupled with a LTQ-orbitrap elite high-resolution mass spectrometer (Thermo-Fisher Scientific, USA) in positive and negative ion modes. For imaging, the AP-MALDI source was operated in a “constant speed raster” motion mode with a stage stepping size of 20 μm. The laser energy was 2000 Hz, and spot size was <20 μm.^41^ Spectrum acquisition: 500 ms maximum injection time; mass range: 350–1600 Da; and mass resolution: 120 k at *m/z* 400.

### MSI: Data Processing and Interpretation

TOF-SIMS data analysis was performed using SurfaceLab 7 (IONTOF GmbH, Germany). MALDI-MSI data were analyzed using Thermo Xcalibur 2.2 and Thermo ImageQuest (Thermo-Fisher Scientific, USA), METASPACE (https://metaspace2020.eu), and LipostarMSI (Molecular Horizons Srl, Italy). All images were normalized to total ion count (TIC) and denoised by hotspot removal. Segmentation (bisecting K-means algorithm), colocalization, and PCA analyses were performed using LipostarMSI.

## Results and Discussion

### PFOA Identification and Localization

**Figure 1:**
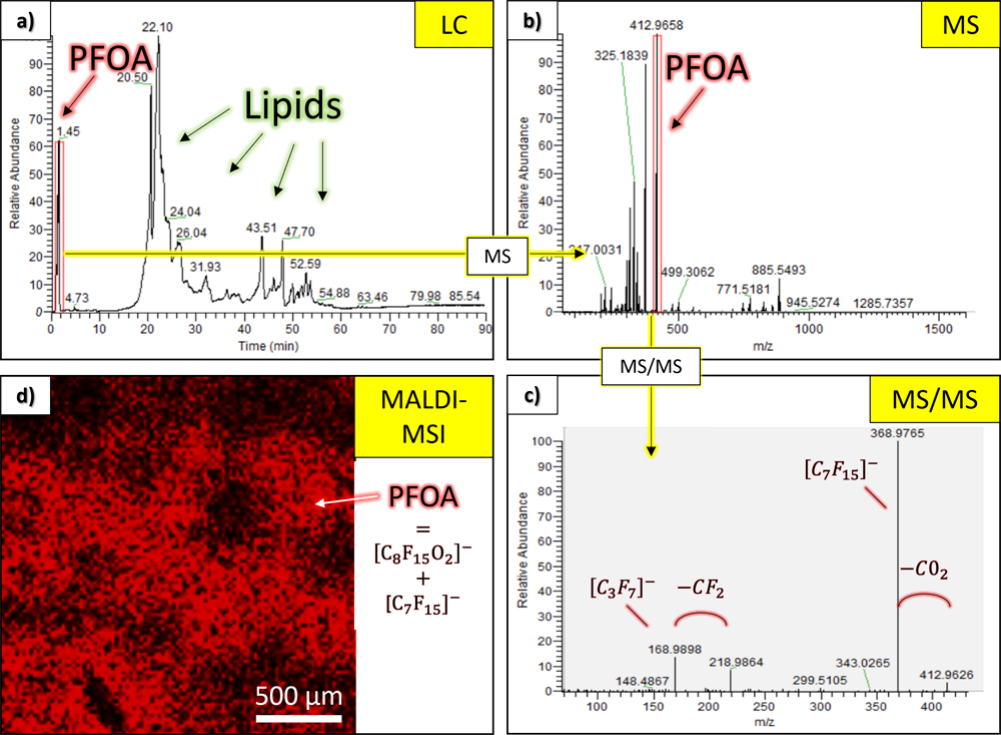
PFOA identification and localization in the PFOA-exposed liver for one representative animal by LC–MS/MS and MALDI-MSI: (a) liquid chromatogram (LC) at 1.45 min, (b) MS spectrum at *m/z* 412.966, (c) MS/MS spectrum, and (d) MALDI-MSI image of PFOA (sum of [C_8_F_15_O_2_]^−^ and [C_7_F_15_]^−^).

PFOA was clearly identified in PFOA-exposed liver samples (“case”) using LC–MS/MS analysis after 1.45 min (Figure 1a) at *m/z* 412.966 (Figure 1b) and confirmed by MS^2^ spectrum (Figure 1c). The experimental isotope pattern of PFOA is consistent with the theoretical one (Figure S1), and the MS^2^ spectrum is in line with the literature.^42^ Due to its omnipresence in the environment (and particularly in the water), we also detected it in the control liver and even in the blank. The PFOA signal intensity was two orders of magnitude lower in the control liver than in the case and two orders of magnitude lower in the blank than in the control (Figure S2). The analytical reproducibility and the biological variability were assessed by normalization with internal standards (Figure S3).

PFOA was also successfully localized inside the liver using MALDI-MSI (Figure 1d) and TOF-SIMS (Figure S4) techniques. The MALDI image reveals a heterogeneous localization of PFOA within the tissue, which could not be observed using a higher-resolution technique such as TOF-SIMS. Surprisingly, we could not detect any PFOA in the blood vessel using both techniques although many studies have reported the presence of PFOA in blood.^43^ However, this could be a vein, an artery, or even a biliary duct, influencing its chemical composition. The PFOA concentration in the blood could also be below the detection limit. Contrary to LC–MS/MS results, PFOA was not detected in the control (images not reported) probably due to the detection limit of the instruments.

### Lipid Annotation and Statistical Analysis

Based on LC–MS/MS data analysis using MS-dial, about 1500 lipid species were identified and 383 identified lipid peaks were significantly different between the case and the control (only MS/MS peaks, *p*-values below 0.05, and absolute value of fold changes higher than 2). Among significant lipids, 171 lipid species were identified in positive mode and 233 lipid species in negative mode. Of those lipids, 21 were identified in both ion modes and for further analysis, they were excluded from the positive data set to avoid duplicates.

**Figure 2:**
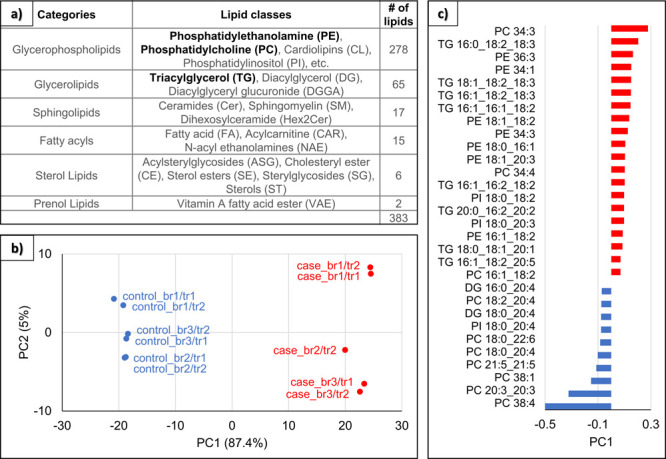
Lipid annotation and statistical analysis. (a) Summary of the LC–MS/MS data of the lipid categories and classes significantly impacted after PFOA exposure. The most impacted lipid classes in terms of lipid numbers appear in bold. (b) Two dimensional PCA scores of lipid species for controls (blue) and cases (red). The label name indicates the biological replicate (first label number) and the technical replicate (second label number). (c) Loading plot showing lipids with the highest absolute PC1 value: the lipids in red are significantly higher in the case samples and the lipids in blue are significantly higher in the control samples.

Figure 2a summarizes all the hepatic lipid categories significantly impacted after PFOA exposure (grouping data of negative and positive modes), some lipid classes (the most impacted appearing in bold), and the number of lipids for each category (see the number of lipids for each lipid class in Table S1). The most represented category is glycerophospholipids. Overall, phosphatidylethanolamine (PE), phosphatidylcholine (PC), and TG are the most impacted lipid classes in terms of lipid number (see lipid structures in Figure S5). All PE, PC, and TG lipid species significantly impacted by PFOA exposure and present in higher or lower levels are listed in Tables S2–S4, respectively. A higher (lower) level of one lipid species in the liver can be the result of either a higher (lower) production or a lower (higher) consumption within the tissue resulting in accumulation (decrease), or even a lower (higher) export into the blood. One interesting observation is that, for example, PE 38:4 | PE 18:0_20:4 is present at lower levels, while PE 38:4 | PE 18:1_20:3 is present at higher levels. Both lipids have the same chain length and number of double bonds, but the latter are positioned differently in the chain, highlighting the importance of precise molecule identification.

The high information content of such global lipid analysis requires the use of data analysis methods, such as multi-variate analysis, which enables the identification of the lipids contributing the most to variation or separation between groups. We first performed PCA analysis, an unbiased method, and then PLS-DA, a supervised method based on PCA results. The PCA scores plot (Figure 2b) shows a clear separation between the control and case groups, confirming the metabolomic difference at the hepatic level and thus allowing for subsequent supervised analysis. The first principal component (PC1) explained 87.4% of the observed variation within the data. A lipid with a positive PC1 value is more abundant in the case while a lipid with a negative PC1 value is more abundant in the control. It should be mentioned that the technical replicates (tr1 and tr2) of all biological replicates (br1, br2, and br3) show very similar values, as expected. The PCA loading plot (Figure 2c) highlights the lipids maximizing the variance (PC1 ≥ 0.07 or ≤ −0.07) between the case and control samples. The PLS-DA analysis, highlighting the lipids that maximize the co-variance, and performed for comparison purposes, shows similar results (Figure S6).

### Lipidomic Pathway Analysis

Based on the LC–MS/MS data, we first performed lipidomic pathway analysis using the MetaboAnalyst online platform as done in similar studies.^22,44^Figure S7 gives an overview of all pathways significantly impacted by PFOA exposure, highlighting the glycerophospholipid metabolism as being the most impacted pathway. This result was expected based on the data displayed in Figure 2a and corroborates previous findings in human and animal studies.^22,44^ Indeed, the glycerophospholipid metabolism pathway was overrepresented in human groups highly exposed to PFAS (including PFOA) and was further correlated in patients with high macrosteatosis and increasing stages of fibrosis.^22^ In addition, this pathway was also overrepresented in the mouse liver after PFOS exposure.^44^ The main drawback of this pathway analysis is that lipid classes (PE, PC, etc.) are mostly considered (Figure S8) without considering the lipid species in each class. However, as observed in Figure 2c, the levels of single lipid species from the same class can be completely different after PFOA exposure (higher or lower in the case samples).

**Figure 3:**
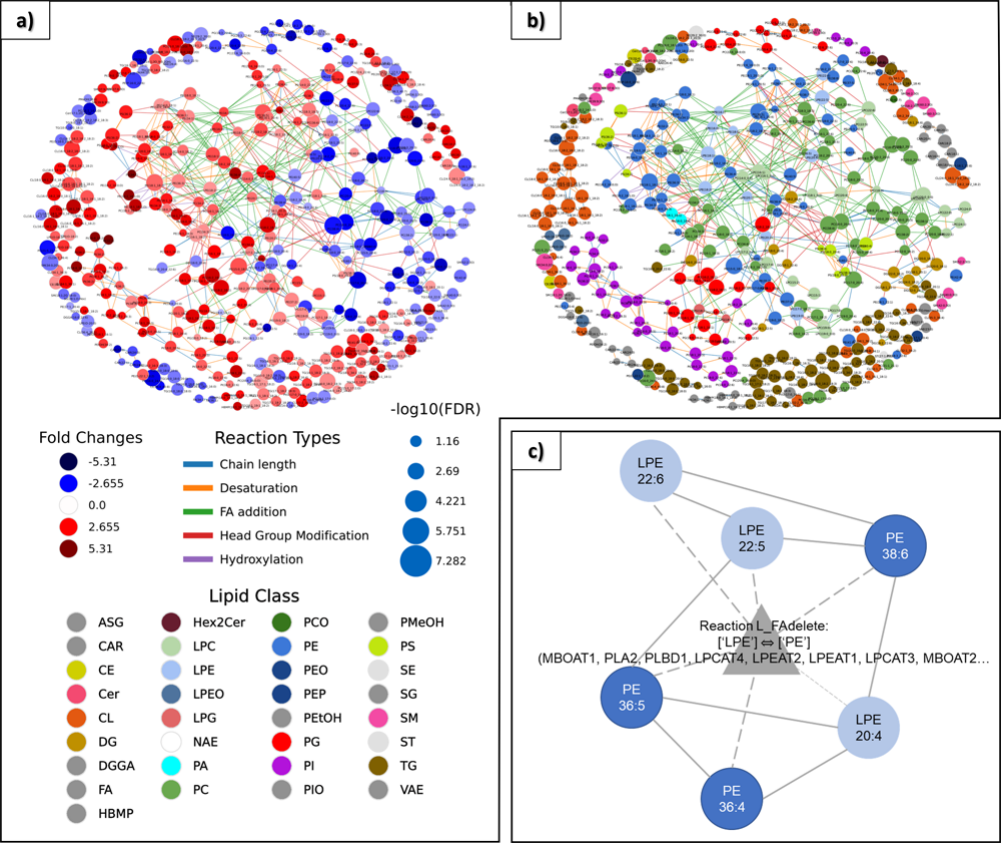
(a,b) LINEX lipid network based on LC–MS/MS lipid data. Each node represents a lipid species, and each edge between a pair of nodes indicates a biochemical reaction transforming the lipid species into each other within one class or between classes. Edge colors indicate the reaction types, and node sizes indicate the negative log10 FDR corrected *p*-values of lipid species between the control and case groups. (a) Node colors represent the log fold change between both groups (red: higher level in the cases and blue: lower level in the cases). (b) Node colors represent the lipid classes. (c) LINEX enrichment network with the LPE to PE reaction at the center (*p*-value of 0.0167). Spherical nodes represent lipids, and the colors refer to LPE (light blue) or PE (dark blue) lipid species. Triangular nodes represent the enzyme class.

Therefore, we instead used LINEX software, providing lipidomic networks connecting lipid species together, as shown in Figure 3a,b. The first network (Figure 3a) shows a global view of the changes in the lipidome between the control and the case, while the second one (Figure 3b) is more focused on the lipid classes impacted by PFOA exposure. In these networks, each node represents a lipid species, and each edge between a pair of lipids indicates the biochemical reaction that transforms one lipid species into another within each class or between classes. Edge colors indicate the reaction types (i.e., chain length or elongation, desaturation, FA addition, head group modification, or hydroxylation), and node sizes indicate the significance of the difference between the PFOA-exposed and control conditions (the larger the nodes are, the more strongly altered the lipids are). In Figure 3a, node colors represent the log fold change between the two conditions (red: higher levels in PFOA-exposed animals and blue: lower levels in PFOA-exposed animals) while in Figure 3b, node colors represent the lipid classes.

Regarding fold changes, there is an equivalent number of lipids with increased or decreased levels after PFOA (Figure 3a). Some categories such as PI or PE have mainly increased levels in PFOA-exposed livers while some others such as PC have mainly decreased levels. The other ones are either increased or decreased. Regarding reaction types, FA addition is the most prevalent one (highlighted in green in Figure 3a,b), which suggests that the pathways involving this type of metabolic reactions are specifically impacted by the PFOA.

Figure 3c shows the enrichment network generated by LINEX based on the global networks. The algorithm highlights a dysregulated subnetwork maximizing the reaction difference between the control and case conditions.^39^ The resulting subnetwork consists only of PE and LPE lipid species, and the LPE to PE class reactions, which can be catalyzed by phospholipase enzymes such as PLA2, PLBD1 (producing FA and LPE from PE), or lysophospholipid acyltransferase enzymes such as MBOAT1, LPCAT4, and LPEAT1 (producing PE by adding one FA to LPE). The specific LPE to PE reaction that seems to be modulated by PFOA involves myristic acid (14:0), palmitic acid (16:0), palmitoleic acid (16:1), or linoleic acid (18:2) as fatty acids. We should further investigate the regulation of such enzymes in order to validate this hypothesis.

**Figure 4:**
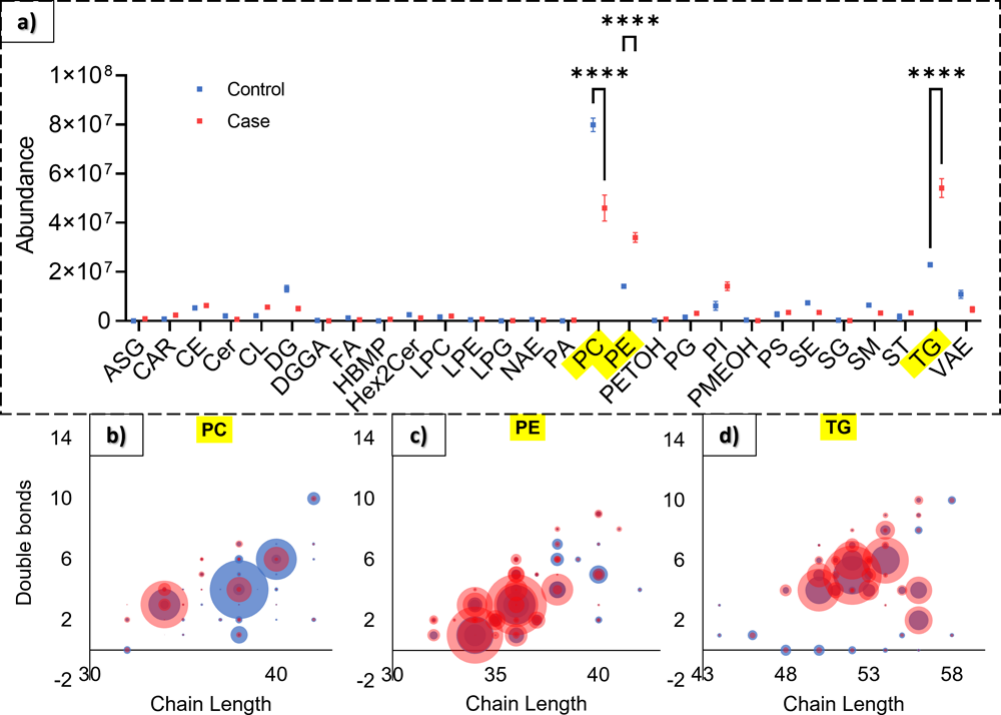
(a) Lipid class composition abundance highlighting PC, PE, and TG classes, which are statistically different between case and control conditions (one-way ANOVA, ^****^p-value < 0.0001) and (b–d) corresponding chain length–double bond abundance. The dot sizes indicate the lipid abundance, and the dot colors correspond to the control (blue) or case (red) samples.

We further explored the lipidomic changes induced by PFOA exposure. Figure 4a represents the abundance (i.e., the average signal intensity of all lipid species) in the control and case groups for each lipid class. The three lipid classes, which are statistically different between both groups, are PC, PE, and TG. The abundance of PC species is significantly higher in control livers, whereas the abundance of PE and TG is significantly in PFOA-exposed livers (*p*-value < 0.0001). PE and PC are the most abundant phospholipids in the mammalian cell membrane. Abnormally high or low cellular PC/PE molar ratios may affect energy metabolism and play a role in the progression from steatosis to steatohepatitis.^45^ Moreover, the relative abundance of PC and PE regulates the size and dynamics of lipid droplets (LDs) (TG reservoir), corroborating the excessive TG accumulation within hepatocytes after PFOA exposure, as concluded from our LC–MS/MS analysis and also reported in many studies.^10−13^

Figure 4b−d shows the double bond abundance depending on the chain length for PC, PE, and TG lipid classes, respectively. The dot sizes indicate the lipid abundance, and the dot colors correspond to the control (blue) or case (red) samples. For example, the PC lipids present at higher levels in control samples have a chain length between 38 and 40 carbons and a double bond number of between 4 and 6 while PC lipids present at higher levels in case samples have a chain length of 34 carbons and three double bonds. Besides PE, PC, and TG, Figure S9 provides a summary of the chain length–double bond relationship for the other most abundant classes. Comparing all classes, the lipids with a longer chain length and a higher number of double bonds tend to decrease with PFOA exposure (i.e., they are more abundant in control samples) and contrariwise and the lipids with a shorter chain length and fewer double bonds tend to increase with PFOA exposure (i.e., they are more abundant in case samples). For example, increased levels of glycerophospholipid containing saturated fat could result in a rigid membrane environment and affect normal functions of membrane proteins.^30^ However, there are some exceptions: for example, most DG lipids decrease whereas most TGs increase after exposure.

In addition, it is worth noting that the lipid species that are mostly less abundant in control samples are made of dietary fatty acids. Indeed, animals are unable to synthesize de novo either *n*-6 or *n*-3 polyunsaturated fatty acids (PUFAs) or any fatty acid with more than three double bonds; instead, they must be obtained from the diet.^46^ However, lipids that are more abundant in the case samples might not come from increased fatty acid synthase (FAS) activity because the FAS expression did not change after exposure (data not shown). Instead, the activity of elongases and desaturases might be disturbed, and their expression should be assessed.

The lack of PUFAs in the liver has been shown to be harmful, e.g., PUFAs have been reported to play a role in protecting against non-alcoholic fatty liver disease and its progression to more severe forms.^47,48^

### Lipid Localization and Statistical Analysis

**Figure 5:**
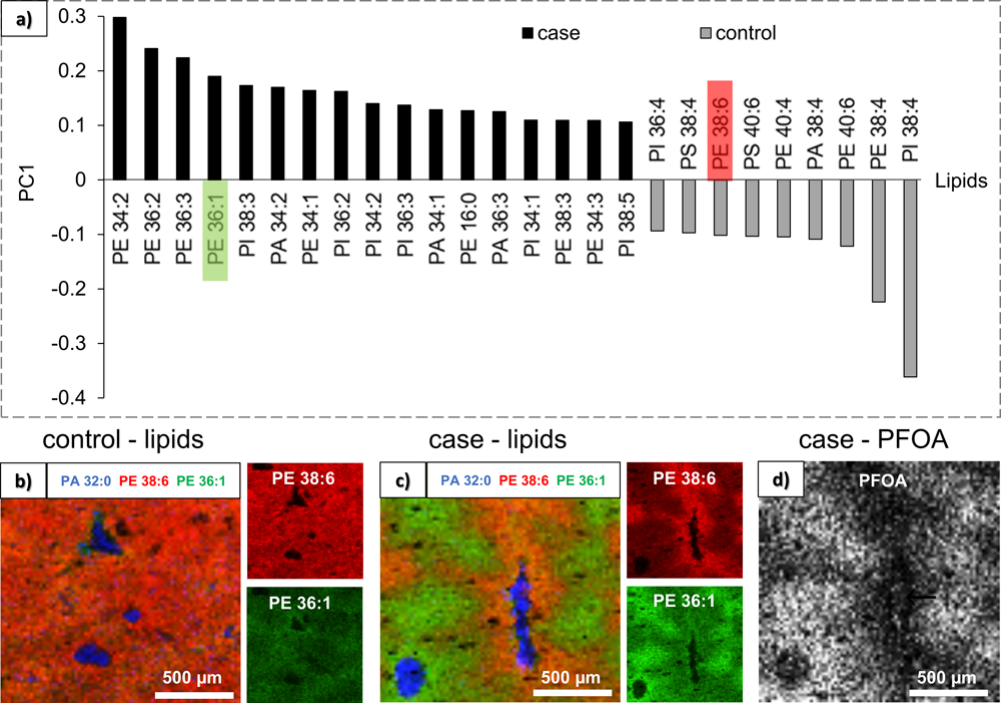
(a) PCA loading plot of the lipids contributing most to the variation (PC1 ≥ 0.1 or ≤ −0.1) between case and control images by MALDI in negative mode. Lipids with a positive PC1 value have increased levels after PFOA exposure (black bars) while lipids with a negative PC1 value have decreased levels after exposure (gray bars). (b,c) MALDI superposition images of PE 38:6 (red), PE 36:1 (green), and PE 32:0 (blue) for the (b) control and (d) case conditions. (d) MALDI images of PFOA (sum of C_8_F_15_O_2_^–^ and C_7_F_15_^–^) for the case condition.

In addition to the lipidomic pathway investigations presented above, lipids were identified and localized in the tissue sections from both groups (two biological replicates per group and two images in each polarity) by MALDI-MSI. PCA analysis was then performed on those lipidomic data on the whole images (Figure S10). As for LC–MS/MS results, PC1 explains most of the differences between the case and control samples. However, the lipids contributing most to the variation were slightly different than the ones identified in LC–MS/MS data using MS-dial, which is discussed below. Figure 5a reveals the lipids with the highest and lowest PC1 values (≥ 0.1 and ≤ −0.1) based on MALDI analysis in negative mode. The LINEX analysis of the LC–MS/MS data showed lipids with 38 and 40 carbons and more than four double bonds to be correlated with the control samples, while shorter, more saturated lipids were correlated with the PFOA exposed samples. The same trend is visible in Figure 5a. Figure 5b,c shows the image superposition of three lipids before and after PFOA exposure: PE 36:1 (increased level), PE 38:6 (decreased level), and PA 32:0 (unchanged level). PA 32:0 localizes in the blood vessels (BVs) and remains unchanged after exposure. On the contrary, PE 36:1 and PE 38:6 distribute evenly within the tissue before PFOA exposure and unevenly after exposure. Indeed, heterogeneities appear in terms of lipid distribution in Figure 5c, and it seems that some regions remain unchanged after exposure (less impacted) while others display changes in lipid levels: PE 36:1 increases and PE 38:6 decreases. Moreover, the BVs are not surrounded by the same regions, probably because they consist of different vascular tissues or even bile ducts. Our most interesting observation was that PFOA localizes in the region the most impacted, which means with a higher level of PE 36:1 and a lower level of PE 38:6 (Figure 5d). The PFOA does not appear in the PCA analysis because it was performed only on identified lipids using the LIPIDMAPS database, thus excluding PFOA ions.

**Figure 6:**
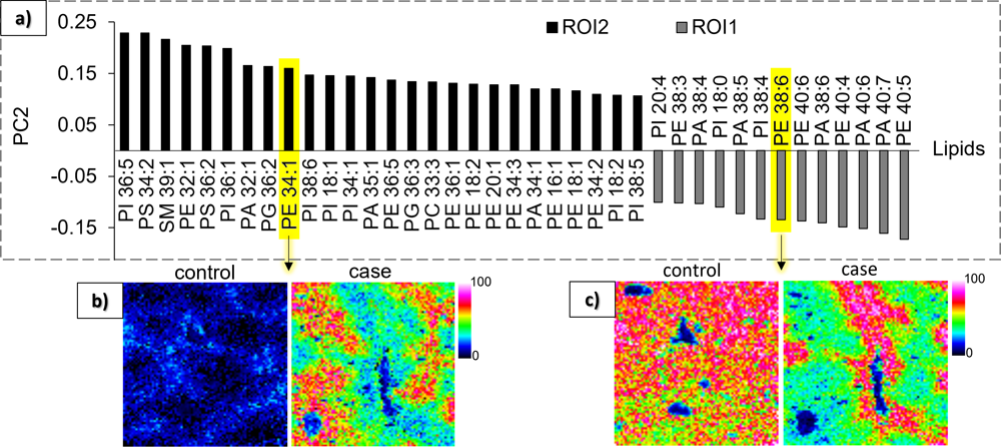
(a) PCA loading plot of the lipids contributing most to variation (PC2 ≥ 0.1 or ≤ −0.1) between ROI1 and ROI2 isolated in the images of the case samples in negative mode. Lipids with a positive PC2 value have higher levels in ROI2 (black bars) while lipids with a negative PC2 value have higher levels in ROI1 (gray bars). (b,c) MALDI images of PE 34:1 (b) and PE 38:6 (c) in control and case samples.

Contrary to LC–MS/MS analysis where lipids are extracted from tissue homogenates, here we can distinguish different regions. We further analyzed the images of the case samples by performing a segmentation (Figure S11), identifying three main regions: the BVs and two regions of interests (ROIs), called ROI1 and ROI2, with different lipidomic profiles. A PCA analysis was then performed on ROI1 and ROI2, which only appear after exposure. PC2 explains the difference between the two ROIs at 22.5%, and Figure 6a reveals the lipids with the highest (related to ROI2) and lowest (related ROI1) PC2 values (≥ 0.1 and ≤ −0.1). The localization of PE 34:1 (Figure 6b) and PE 38:6 (Figure 6c) in control and case samples are shown as representative examples of lipids related to ROI2 and ROI1, respectively. For both examples, we observed that the signal intensity in ROI1 in the case is similar to the one in the control, which is probably less impacted by PFOA exposure than ROI2. Indeed, lipids related to control samples in Figure 5a are also related to ROI1 in Figure 6a, such as PI 38:4, PE 40:6, PA 38:4, PE 40:4, or PE 38:6. On the contrary, the lipids related to case samples are mostly related to ROI2, such as PE 34:1, PI 34:1, or PE 36:1. Segmentation and statistical analysis were also performed on the positive mode images (Figure S12). By identifying and localizing lipids detected in both modes, such as PE 38:3, each ROI obtained from the segmentation in positive mode could be correlated with the corresponding one in negative mode. In addition, BV regions were clearly identified by the heme (from the hemoglobin), which is only detected in positive mode.

Although, as mentioned above, PFOA does not appear in the PCA analysis, it is also localized in ROI2. Indeed, the colocalization analysis performed on the whole data set clearly showed that a significant, non-random spatial correlation exists between PFOA and lipids, such as PE 36:1, PE 34:1, and PA 34:1 identified in ROI2. In conclusion, PFOA is mostly localized in the ROI with shorter chain, more saturated lipids.

Comparing MALDI and LC–MS/MS results, the levels of lipid species after PFOA exposure change similarly (increase or decrease) for both techniques, as expected (Table S5). However, due to differences in lipid extraction (LC → solvents, MALDI → matrix) and ionization (LC → ESI, MALDI → laser), a few lipids present in the MSI data could not be identified with LC–MS/MS and conversely, many lipids identified by LC–MS/MS were not detected/identified by MALDI, emphasizing the importance of combining techniques to have a complete picture of lipidomic profiles and their changes after PFOA exposure. In this regard, only a few TG lipids were identified by MALDI in positive mode. Likewise, the LDs, intracellular energy stores mainly compartmentalizing TG lipids, cannot be observed on MALDI images. However, an increase of the number and size of those LDs after PFOA exposure was observed by LM using Oil Red O (Figure S13) and was confirmed in other studies.^11,19^ This issue may be due to problems during sample preparation or the unsuitability of the DAN matrix for the detection of TGs. Methods using salt doping combined to matrix sublimation^49^ or silver-assisted laser desorption ionization^50^ could improve the detection of TG and other neutral lipids.

## Conclusions

This multi-modal MS analysis has provided insightful information about the fate and lipidomic impacts of PFOA in the mouse liver. The perflurinated compound was identified with all the MS techniques. Regarding lipidomics, LC–MS/MS analysis enables us to identify a great range and number of lipid species but only gives a general overview of the changes between sample groups, while MALDI-MSI analysis has the advantage of providing location-specific lipidomic profiles within liver tissues. Due to its destructive nature, TOF-SIMS mostly detects fatty acids (fragments) and entire lipid molecules with very low intensities. As one fragment can be associated with many different molecules, lipid imaging with this technique was not further investigated in this paper.

High-dose acute exposure to PFOA leads to hepatic lipid dysmetabolism, such as the impairment of glycerophospholipid or glycerolipid metabolism. These metabolism pathways were also impacted in previous human and animal studies with PFAS. Rodent studies with PFAS (usually at high doses) have shown increased intrahepatic lipid (mainly TG) concentrations. Similarly, this study has demonstrated increased intrahepatic TG levels but has also shown the dysregulation of other lipid classes, such as PC and PE. The relative abundance of PC and PE lipids regulates the size and dynamics of LDs (TG reservoir), corroborating the excessive TG accumulation within hepatocytes after PFOA exposure. In addition, we have demonstrated the importance of considering the lipids as individual lipid species and not according to their lipid class. Indeed, lipid species in the same lipid class can behave completely differently, even those with the same chain length and number of double bonds but different distributions between the two fatty acid chains. Therefore, a precise lipid identification is essential for a rigorous lipidomic pathway analysis.

LINEX network analysis has provided an overview of the changes of the lipidome after PFOA exposure, and network enrichment analysis showed that the LPE to PE class reactions, which can be catalyzed by enzymes such as MBOAT1, were the most impacted subnetwork. Enzyme regulation analysis or the targeted transcriptomic measurement of genes and proteins could help validate and aid the interpretation of these lipidomic data.

Comparing all lipid classes, lipids with a longer chain length and a higher number of double bonds tend to decrease with PFOA exposure and contrariwise, the lipids with shorter chain length and less double bonds tend to increase with PFOA exposure. It is noteworthy that the lipid species most impacted by PFOA exposure are made exclusively from dietary fatty acids (4+ double bonds) while lipids increased in case samples can be derived from either dietary sources or de novo fatty acid synthesis.

Although cross-sections of the liver show a homogeneous patchwork of hepatocytes infiltrated with some vascular tissue and bile ducts, the lipid imaging analysis revealed different regions with their own lipidomic profile. PFOA was localized in one of those regions, colocalizing with some dysregulated lipid species (shorter chain, more saturated lipids). Analytical development enabling an MS^2^ acquisition every other pixel as demonstrated by Ellis et al.^51^ could provide a precise lipid identification and localization and would definitely be a step forward for lipidomics.

## Supporting Information Available

PFOA isotope pattern; PFOA signal intensity evaluation by LC–MS; PFOA peak normalization by internal standards; PFOA localization inside PFOA-exposed liver by TOF-SIMS; chemical structure of fatty acyls, glycerolipids, and glycerophospholipids; PLS-DA analysis of lipid species from LC–MS/MS data; metabolomic pathways significantly impacted in mouse liver after PFOA exposure; glycerophospholipid metabolism; chain length–double bond abundance for the main lipid classes; PCA score plot of lipid species identified by MALDI in negative and positive modes; principle of image segmentation; PCA loading plots of lipid species identified by MALDI in positive mode; visualization of LDs in case sample by LM using Oil-Red-O; lipid classes significantly impacted after PFOA exposure from LC–MS/MS data; PE lipid species significantly impacted after PFOA exposure from LC–MS/MS data; PC lipid species significantly impacted after PFOA exposure from LC–MS/MS data; TG lipid species significantly impacted after PFOA exposure from LC–MS/MS data; and comparative study between MALDI and LC–MS/MS results (PDF)
