# Supplementary material for: Lipidomic Profiling of PFOA-Exposed Mouse Liver by Multi-Modal Mass Spectrometry Analysis
Source: Anal Chem. 2023 Apr 7;95(16):6568–76. doi: 10.1021/acs.analchem.2c05470 (PMC10134131; doi:10.1021/acs.analchem.2c05470)
Supplement: Supplementary file 1 — ac2c05470_si_001.pdf [file ac2c05470_si_001.pdf]

Supporting Information

Lipidomic profiling of PFOA-exposed mouse liver by multi-modal mass spectrometry analysis

Charlotte B. A. Stoffels[*1,2], Tina B. Angerer[#1], Hervé Robert[3], Nathalie Poupin[3], Laila Lakhal[3], Gilles Frache[1], Muriel Mercier-Bonin[3] and Jean-Nicolas Audinot[1]

[1]Department of Materials Research and Technology, Luxembourg Institute of Science and Technology, Belvaux, 4422, Luxembourg
[2]Faculty of Science, Technology and Medicine, University of Luxembourg, Esch-sur-Alzette, 4365, Luxembourg
[3]Toxalim, Université de Toulouse, INRAE, INP-ENVT, INP-EI-Purpan, Université de Toulouse 3 Paul Sabatier, Toulouse, 31027, France

#Present address: Department of Pharmaceutical Biosciences, Uppsala University, Uppsala, 751 05, Sweden
*Corresponding author: charlotte.stoffels@list.lu

TABLE OF FIGURES

Figure S1. PFOA isotope pattern S2
Figure S2. PFOA signal intensity evaluation by LC-MS....S2
Figure S3. PFOA peak normalization by internal standards....S3
Figure S4. PFOA localization inside PFOA-exposed liver by TOF-SIMS....S3
Figure S5. Chemical structure of fatty acyls, glycerolipids and glycerophospholipids....S5
Figure S6. PLS-DA analysis of lipid species from LC-MS/MS data....S9
Figure S7. Metabolomic pathways significantly impacted in mouse liver after PFOA exposure....S9
Figure S8. Glycerophospholipid metabolism....S10
Figure S9. Chain length – double bond abundance for the main lipid classes....S10
Figure S10. PCA score plot of lipid species identified by MALDI in negative and positive modes....S11
Figure S11. Principle of image segmentation....S11
Figure S12. PCA loading plots of lipid species identified by MALDI in positive mode....S11
Figure S13. Visualization of lipid droplets in case sample by light microscopy using Oil-Red-O....S13

TABLE OF TABLES

Table S1. Lipid classes significantly impacted after PFOA exposure from LC-MS/MS data....S4
Table S2. PE lipid species significantly impacted after PFOA exposure from LC-MS/MS data....S6
Table S3. PC lipid species significantly impacted after PFOA exposure from LC-MS/MS data....S7
Table S4. TG lipid species significantly impacted after PFOA exposure from LC-MS/MS data....S8
Table S5. Comparative study between MALDI and LC-MS/MS results....S12

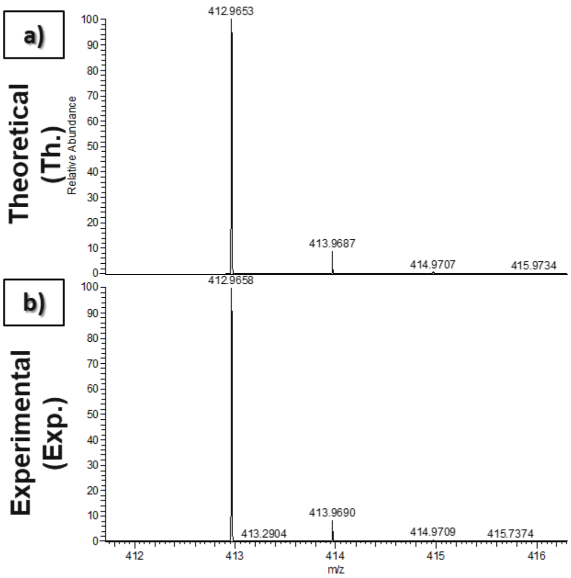

Exp. m/z	Th. m/z	Δm/m (ppm)	Exp. Isotope pattern	Th. Isotope pattern
412.9658	412.9653	-1.2	100.00%	100.00%
413.9690	413.9687	-0.7	8.21%	8.73%
414.9709	414.9707	-0.5	0.60%	0.74%

Figure S1. PFOA isotope pattern: comparison between a) the theoretical (th.) and b) the experimental (exp.) isotope pattern of PFOA (simulation performed with Thermo Xcalibur Qual Browser in profile mode at 60000 resolving power).

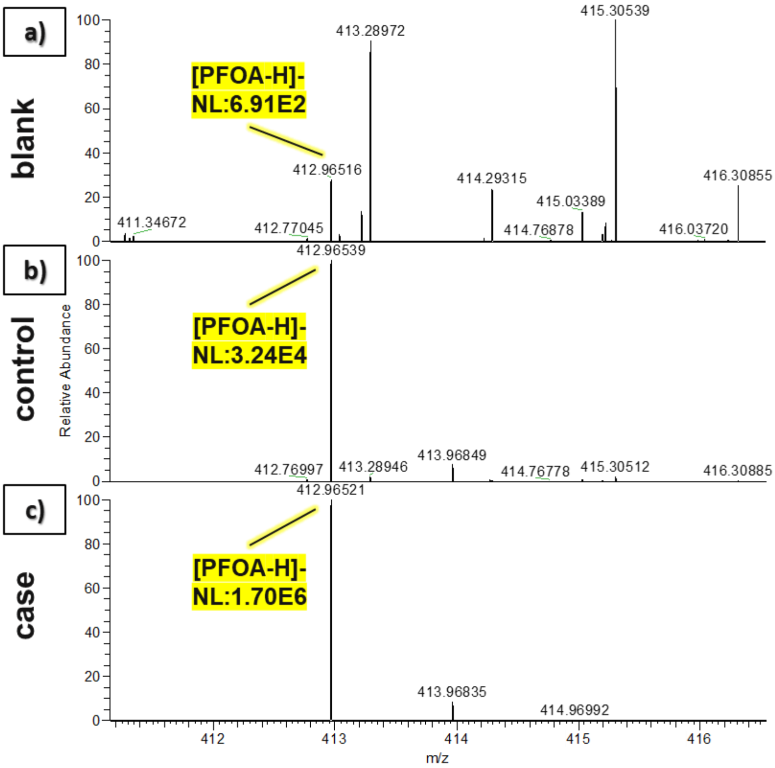

Figure S2. PFOA signal intensity evaluation by LC-MS (at m/z 412.966): comparison between a) the blank, b) the control and c) the case for one representative sample.

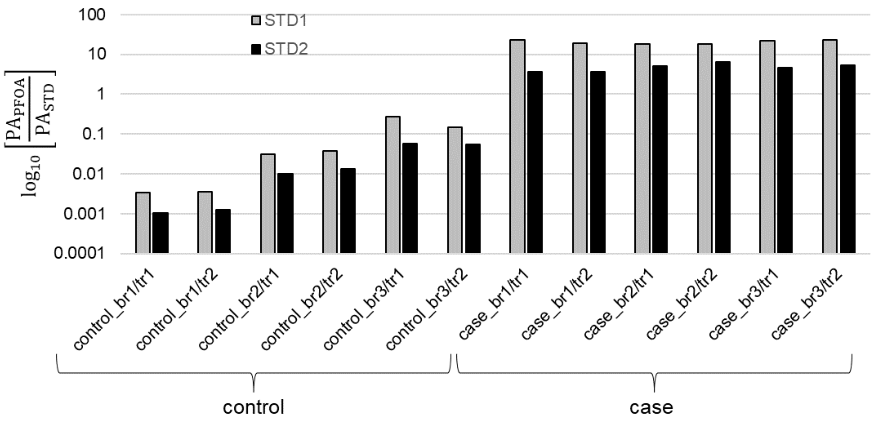

Figure S3. PFOA peak (*m/z* 412.9658) normalization by two internal standards: 15:0-18:1(d7) PE (STD1, *m/z* 709.55) and C15 Ceramide-d7 (STD2, *m/z* 529.53) in control and case samples (PA = peak area).

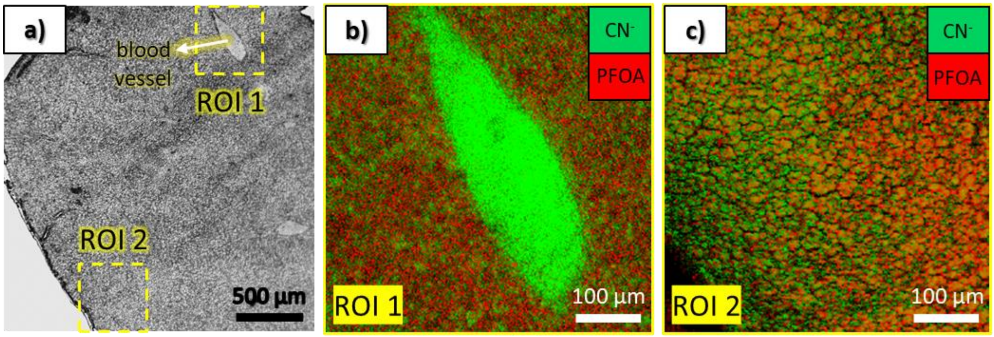

Figure S4. PFOA localization inside PFOA-exposed liver: a) light microscopy image, b) and c) TOF-SIMS images of two regions of interest (ROIs) showing PFOA localization (sum of F^-, $C_7F_{15}^-$ and $C_8F_{15}O_2^-$ $in\ red$) and CN- (in green) that provides morphological information. Images for one representative animal are given.

Table S1. Summary from LC-MS/MS data of all the lipid classes significantly impacted after PFOA exposure. The most impacted lipid classes in terms of lipid amounts appear in bold. Lipid classes with species that are present in lower or higher levels (level change), or both (in liver of treated compared to control animals) are highlighted in black, grey or white, respectively.

Abbreviation	Lipid class	Main lipid class	Categories	# of lipids	Level change
FA	Free fatty acid	Fatty acids and Conjugates [FA01]	Fatty acyls [FA]	3	lower
CAR	Acylcarnitine	Fatty esters [FA07]	Fatty acyls [FA]	11	higher/lower
NAE	N-acyl ethanolamines	Fatty amides [FA08]	Fatty acyls [FA]	1	lower
DGGA	Diacylglyceryl glucuronide	Other Glycerolipids [GL00]	Glycerolipids [GL]	1	lower
DG	Diacylglycerol	Diradylglycerols [GL02]	Glycerolipids [GL]	15	lower
**TG**	**Triacylglycerol**	**Triradylglycerols [GL03]**	**Glycerolipids [GL]**	**49**	**higher/lower**
PetOH	Phosphatidylethanol	Other Glycerophospholipids [GP00]	Glycerophospholipids [GP]	2	higher
PMeOH	Phosphatidylmethanol	Other Glycerophospholipids [GP00]	Glycerophospholipids [GP]	1	lower
LPC	Lysophophatidylcholine	Glycerophosphocholines [GP01]	Glycerophospholipids [GP]	21	higher/lower
**PC**	**Phosphatidylcholine**	**Glycerophosphocholines [GP01]**	**Glycerophospholipids [GP]**	**57**	**higher/lower**
LPE	Lysophosphatidylethanolamine	Glycerophosphoethanolamines [GP02]	Glycerophospholipids [GP]	15	higher/lower
**PE**	**Phosphatidylethanolamine**	**Glycerophosphoethanolamines [GP02]**	**Glycerophospholipids [GP]**	**64**	**higher/lower**
PS	Phosphatidylserine	Glycerophosphoserines [GP03]	Glycerophospholipids [GP]	9	higher/lower
HBMP	Hemibismonoacylglycerophosphate	Glycerophosphoglycerols [GP04]	Glycerophospholipids [GP]	3	higher
LPG	Lysophosphatidylglycerol	Glycerophosphoglycerols [GP04]	Glycerophospholipids [GP]	2	higher
PG	Phosphatidylglycerol	Glycerophosphoglycerols [GP04]	Glycerophospholipids [GP]	24	higher/lower
PI	Phosphatidylinositol	Glycerophosphoinositols [GP06]	Glycerophospholipids [GP]	38	higher/lower
PA	Phosphatidic acid	Glycerophosphates [GP10]	Glycerophospholipids [GP]	2	higher
CL	Cardiolipins	Glycerophosphoglycerophosphoglycerols [GP12]	Glycerophospholipids [GP]	40	higher/lower
VAE	Vitamin A fatty acid ester	Isoprenoids [PR01]	Prenol Lipids [PR]	2	lower
Cer	Ceramides	Ceramides [SP02]	Sphingolipids [SP]	3	higher/lower
SM	Sphingomyelin	Phosphosphingolipids [SP03]	Sphingolipids [SP]	13	higher/lower
Hex2Cer	Dihexosylceramide	Neutral glycosphingolipids [SP05]	Sphingolipids [SP]	1	lower
ASG	Acylsterylglycosides	Other Sterol lipids [ST00]	Sterol Lipids [ST]	1	higher
CE	Cholesteryl ester	Sterols [ST01]	Sterol Lipids [ST]	2	higher/lower
SE	Sterol esters	Sterols [ST01]	Sterol Lipids [ST]	1	lower
SG	Sterylglycosides	Sterols [ST01]	Sterol Lipids [ST]	1	lower
ST	Sterols	Steroid conjugates [ST05]	Sterol Lipids [ST]	1	higher
				383	

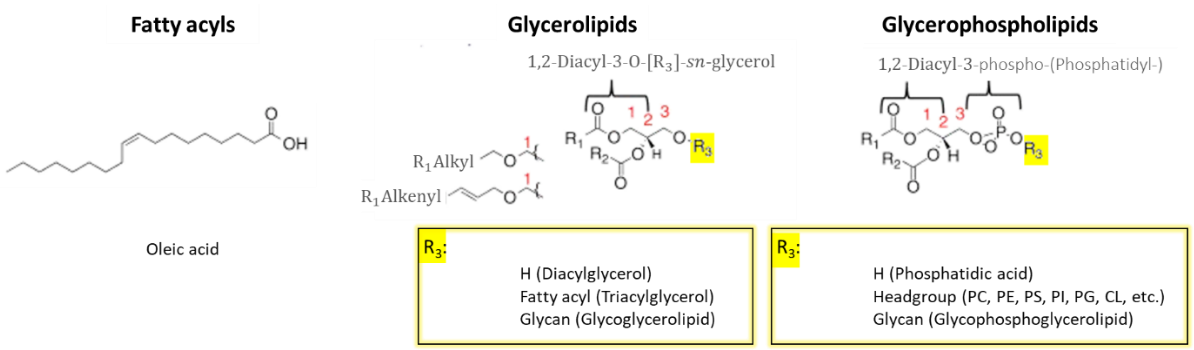

Figure S5. Chemical structure of three major lipid categories: fatty acyls, glycerolipids and glycerophospholipids [1]

Table S2. PE lipid species significantly impacted after PFOA exposure from LC-MS/MS data. Lipid species that are present at higher or lower levels (level change) are highlighted in black or grey, respectively.

RT (min)	*m/z*	Metabolite name	S/N	ANOVA	Fold change	Level change
25.32782	688.4914	PE 32:1|PE 14:0_18:1	6293	4.88E-02	2.3	higher
23.91443	688.4911	PE 32:2	268	9.65E-04	5.3	higher
23.96145	686.4749	PE 32:2|PE 14:0_18:2	2884	2.25E-03	6.8	higher
24.83773	700.4904	PE 33:2|PE 15:0_18:2	331	2.50E-07	3.4	higher
26.75682	718.5375	PE 34:1	12321	1.31E-05	2.3	higher
19.09288	748.5110	PE 34:1;2O|PE 16:0_18:1;2O	38	9.82E-06	16.7	higher
26.78167	716.5226	PE 34:1|PE 18:0_16:1	28147	1.15E-04	2.6	higher
20.66701	730.5004	PE 34:2;O|PE 16:0_18:2;O	67	2.24E-03	13.7	higher
20.32126	730.5002	PE 34:2;O|PE 18:2_16:0;O	35	7.23E-03	5.2	higher
35.58345	714.5067	PE 34:2|PE 16:0_18:2	29	5.78E-04	6.2	higher
26.34176	714.5065	PE 34:2|PE 16:1_18:1	508	1.10E-05	4.1	higher
24.16712	714.5070	PE 34:3	3501	2.94E-05	5.8	higher
24.20536	712.4919	PE 34:3|PE 16:1_18:2	29025	2.47E-04	5.3	higher
22.93782	710.4744	PE 34:4|PE 16:1_18:3	648	2.06E-02	5.6	higher
21.85198	754.5511	PE 35:1	210	2.95E-02	20.9	higher
26.40254	730.5380	PE 35:2	1223	5.70E-05	2.8	higher
26.38845	728.5226	PE 35:2|PE 17:0_18:2	15662	3.00E-06	2.6	higher
28.32805	768.5516	PE 36:1	91	3.98E-03	3.3	higher
28.10963	744.5530	PE 36:1|PE 18:0_18:1	2349	1.71E-06	2.1	higher
35.59570	742.5378	PE 36:2|PE 18:0_18:2	13	6.47E-06	4.7	higher
25.28013	742.5375	PE 36:2|PE 18:1_18:1	195	8.24E-04	2.4	higher
25.85191	742.5385	PE 36:3	12280	2.12E-06	2.4	higher
20.62042	756.5159	PE 36:3;O|PE 18:1_18:2;O	30	4.69E-06	21.5	higher
20.29276	756.5176	PE 36:3;O|PE 18:2_18:1;O	30	3.89E-04	28.1	higher
26.33391	740.5224	PE 36:3|PE 18:0_18:3	2028	3.69E-03	2.7	higher
25.87997	740.5223	PE 36:3|PE 18:1_18:2	19721	1.78E-10	3.0	higher
19.84260	740.5233	PE 36:4	13	1.37E-04	20.4	higher
24.58882	738.5082	PE 36:4|PE 18:2_18:2	7276	7.14E-06	4.3	higher
23.32642	738.5053	PE 36:5	472	1.58E-08	3.4	higher
24.32388	736.4905	PE 36:5|PE 16:1_20:4	4659	6.84E-03	2.6	higher
23.34598	736.4897	PE 36:5|PE 18:2_18:3	1436	1.17E-06	3.6	higher
22.68962	736.4905	PE 36:6	182	3.11E-02	4.9	higher
23.20034	734.4748	PE 36:6|PE 14:0_22:6	618	2.92E-04	2.7	higher
22.70084	734.4747	PE 36:6|PE 16:1_20:5	823	3.36E-02	4.5	higher
27.77959	758.5700	PE 37:2	317	1.37E-06	2.3	higher
27.80562	756.5527	PE 37:2|PE 19:0_18:2	2429	1.45E-05	2.2	higher
21.23408	752.5225	PE 37:5	68	8.41E-05	8.4	higher
24.82367	750.5057	PE 37:5|PE 17:1_20:4	316	7.86E-03	-2.2	lower
23.90221	768.5535	PE 38:4	416	7.49E-05	-3.3	lower
38.90926	766.5393	PE 38:4|PE 18:0_20:4	5	7.04E-03	-2.1	lower
26.32049	766.5370	PE 38:4|PE 18:1_20:3	7559	3.88E-03	3.8	higher
22.00772	812.4971	PE 38:5;3O|PE 20:5_18:0;3O	118	2.48E-02	3.0	higher
23.95860	764.5225	PE 38:6	71	2.51E-02	15.2	higher
20.71121	778.5018	PE 38:6;O|PE 18:1_20:5;O	100	3.63E-04	4.5	higher
20.07516	778.4996	PE 38:6;O|PE 22:6_16:0;O	96	8.70E-04	4.9	higher
24.03403	762.5074	PE 38:6|PE 16:1_22:5	457	2.13E-02	2.6	higher
20.56215	762.5072	PE 38:7	12	2.59E-03	4.7	higher
22.32042	760.5108	PE 38:8	37	4.87E-05	4.6	higher
22.27716	758.4747	PE 38:8|PE 16:2_22:6	76	1.95E-04	3.6	higher
25.81244	776.5228	PE 39:6|PE 17:0_22:6	684	1.42E-04	-2.4	lower
29.27100	800.6162	PE 40:2	101	8.74E-03	-2.8	lower
29.30153	798.6016	PE 40:2|PE 18:1_22:1	219	6.41E-03	-3.1	lower
27.40963	794.5683	PE 40:5	1669	1.52E-05	-2.3	lower
27.43588	792.5504	PE 40:5|PE 18:0_22:5	586	7.12E-05	-2.1	lower
24.76197	788.5219	PE 40:7|PE 20:3_20:4	268	8.86E-03	-2.0	lower
22.54577	786.5061	PE 40:9	220	4.89E-03	2.1	higher
22.55568	784.4902	PE 40:9|PE 18:3_22:6	953	2.97E-03	2.5	higher
21.04752	802.5377	PE 41:8	90	2.89E-05	4.3	higher
28.77364	824.6149	PE 42:4	120	4.28E-05	-2.8	lower
27.23002	730.5765	PE O-36:1|PE O-18:0_18:1	60	5.59E-05	-2.9	lower
27.10547	736.5293	PE O-37:5|PE O-17:1_20:4	170	6.03E-05	-2.1	lower
25.92647	746.5107	PE O-38:7|PE O-16:1_22:6	6314	3.70E-06	-3.7	lower
29.35955	782.6066	PE O-40:3|PE O-22:1_18:2	404	1.23E-04	3.4	higher
25.89810	748.5303	PE P-38:6|PE P-16:0_22:6	871	2.48E-08	-3.9	lower

Table S3. PC lipid species significantly impacted after PFOA exposure from LC-MS/MS data. Lipid species that are present at higher or lower levels (level change) are highlighted in black or grey, respectively.

RT (min)	*m/z*	Metabolite name	S/N	ANOVA	Fold change	Level change
26.01252	792.5756	PC 32:0|PC 16:0_16:0	1187	9.24E-06	-2.2	lower
24.43872	804.5267	PC 32:2;O|PC 15:0_17:2;O	45	1.23E-03	2.1	higher
23.26085	788.5437	PC 32:2|PC 14:0_18:2	2871	2.13E-04	2.7	higher
23.93389	802.5129	PC 32:3;O|PC 18:2_14:1;O	225	7.77E-06	-9.4	lower
21.91829	786.5266	PC 32:3|PC 16:1_16:2	28	1.05E-04	5.8	higher
18.41592	850.5781	PC 34:1;2O|PC 16:0_18:1;2O	11	3.76E-09	6.9	higher
35.08351	816.5746	PC 34:2	21	4.87E-04	4.0	higher
17.06292	848.5635	PC 34:2;2O|PC 16:0_18:2;2O	5	6.35E-05	4.2	higher
19.68024	832.5671	PC 34:2;O|PC 18:2_16:0;O	41	6.68E-04	2.4	higher
35.57634	816.5732	PC 34:2|PC 16:1_18:1	12	9.42E-05	3.0	higher
23.51007	756.5528	PC 34:3	15466	9.98E-05	2.2	higher
23.52825	814.5599	PC 34:3|PC 16:1_18:2	20287	3.82E-07	2.1	higher
22.24639	754.5374	PC 34:4	1587	1.15E-05	7.5	higher
24.09324	828.5300	PC 34:4;O|PC 18:2_16:2;O	36	4.09E-04	-3.4	lower
22.26079	812.5410	PC 34:4|PC 16:1_18:3	256	2.01E-06	6.5	higher
27.93133	848.5916	PC 35:1;O|PC 18:1_17:0;O	378	5.08E-06	-2.3	lower
25.80913	830.5904	PC 35:2|PC 17:0_18:2	457	4.12E-06	-3.3	lower
24.44465	828.5739	PC 35:3|PC 17:1_18:2	786	8.37E-06	2.5	higher
23.91999	826.5592	PC 35:4|PC 15:0_20:4	558	1.08E-05	-2.7	lower
25.81098	842.5906	PC 36:3|PC 18:0_18:3	1587	6.55E-07	-5.5	lower
19.39327	856.5703	PC 36:4;O|PC 20:4_16:0;O	9	2.63E-02	-2.8	lower
22.63095	840.5632	PC 36:4|PC 16:1_20:3	315	1.28E-03	2.2	higher
22.61835	802.5348	PC 36:5	125	9.54E-03	2.1	higher
22.63336	838.5585	PC 36:5|PC 18:2_18:3	1377	1.21E-03	2.2	higher
21.61146	778.5373	PC 36:6	175	1.44E-05	2.7	higher
22.01039	836.5419	PC 36:6|PC 16:1_20:5	287	7.24E-03	4.0	higher
21.31044	836.5414	PC 36:6|PC 18:3_18:3	63	3.93E-07	2.6	higher
27.29549	858.6207	PC 37:2|PC 19:0_18:2	5441	1.93E-05	-2.5	lower
25.57491	854.5889	PC 37:4|PC 17:0_20:4	950	9.12E-10	-7.6	lower
27.70612	838.6309	PC 38:1	3202	6.40E-06	-9.6	lower
28.73789	874.6531	PC 38:1|PC 18:0_20:1	537	1.44E-04	-2.5	lower
27.91385	836.6140	PC 38:2	79	2.33E-07	-2.1	lower
27.92255	872.6368	PC 38:2|PC 20:0_18:2	5993	3.07E-06	-2.5	lower
27.33176	870.6200	PC 38:3|PC 18:0_20:3	1220	2.35E-05	-4.3	lower
26.33573	810.6002	PC 38:4	21680	1.37E-10	-5.4	lower
26.36312	868.6059	PC 38:4|PC 18:0_20:4	43725	3.49E-09	-5.9	lower
25.33904	866.5901	PC 38:5|PC 18:0_20:5	3431	1.22E-06	-3.2	lower
18.91334	880.5666	PC 38:6;O|PC 22:6_16:0;O	4	6.70E-03	-2.5	lower
23.78176	864.5743	PC 38:6|PC 18:1_20:5	1400	2.64E-04	-3.0	lower
23.64483	864.5753	PC 38:6|PC 18:2_20:4	15724	1.11E-07	-6.3	lower
22.79839	862.5568	PC 38:7|PC 16:1_22:6	3479	5.82E-04	2.2	higher
27.09452	882.6199	PC 39:4|PC 19:0_20:4	73	2.99E-06	-8.9	lower
29.14058	900.6672	PC 40:2|PC 22:0_18:2	620	3.17E-04	-3.4	lower
28.22603	898.6519	PC 40:3|PC 20:0_20:3	958	1.41E-04	-2.7	lower
27.31774	896.6349	PC 40:4|PC 18:0_22:4	465	4.45E-07	-3.9	lower
27.72504	896.6340	PC 40:4|PC 20:0_20:4	1188	1.60E-07	-9.9	lower
26.84511	894.6179	PC 40:5|PC 18:0_22:5	394	1.01E-04	-12.3	lower
25.30034	892.6049	PC 40:6|PC 16:0_24:6	2716	3.10E-10	-8.1	lower
25.93522	892.6052	PC 40:6|PC 18:0_22:6	54744	7.45E-10	-7.0	lower
25.90431	834.6001	PC 40:6|PC 20:3_20:3	19949	6.27E-07	-2.7	lower
24.06445	890.5905	PC 40:7|PC 20:3_20:4	710	4.92E-07	-5.5	lower
22.79338	912.5714	PC 42:10|PC 20:4_22:6	1336	3.14E-05	-5.4	lower
22.77089	854.5692	PC 42:10|PC 21:5_21:5	3867	8.88E-08	-5.6	lower
30.22449	870.6944	PC 42:2|PC 21:1_21:1	544	5.65E-04	-2.0	lower
27.34726	920.6344	PC 42:6|PC 20:0_22:6	597	4.80E-08	-6.1	lower
21.92254	878.5748	PC 44:12|PC 22:6_22:6	930	1.58E-02	2.0	higher
28.03302	846.6364	PC O-42:7	131	2.21E-02	-2.1	lower

Table S4. TG lipid species significantly impacted after PFOA exposure from LC-MS/MS data. Lipid species that are present at higher or lower levels (level change) are highlighted in black or grey, respectively.

RT (min)	*m/z*	Metabolite name	S/N	ANOVA	Fold change	Level change
33.91050	766.6920	TG 44:1|TG 10:0_16:0_18:1	133	4.81E-02	-3.6	lower
32.64262	762.6606	TG 44:3|TG 8:0_18:1_18:2	77	3.32E-02	-2.3	lower
34.56133	794.7233	TG 46:1|TG 14:0_16:0_16:1	1221	3.96E-02	-2.8	lower
35.69885	824.7703	TG 48:0|TG 16:0_16:0_16:0	1871	1.74E-02	-3.8	lower
33.50493	816.7066	TG 48:4|TG 14:0_16:1_18:3	584	1.94E-03	3.3	higher
36.26770	852.8020	TG 50:0|TG 16:0_16:0_18:0	1462	1.44E-03	-3.4	lower
34.14595	844.7389	TG 50:4|TG 16:1_16:1_18:2	15552	2.72E-04	2.5	higher
33.62525	842.7219	TG 50:5|TG 16:1_16:2_18:2	8849	4.00E-05	4.7	higher
33.10953	840.7063	TG 50:6|TG 16:1_18:2_16:3	509	2.11E-02	2.4	higher
31.86660	854.6881	TG 50:7;1O|TG 15:1_17:3_18:3;1O	52	4.60E-05	-2.4	lower
32.75871	838.6910	TG 50:7|TG 16:1_18:2_16:4	45	5.97E-04	5.6	higher
34.51642	858.7548	TG 51:4|TG 16:1_17:1_18:2	1521	2.33E-06	2.1	higher
33.98355	856.7404	TG 51:5|TG 15:0_18:2_18:3	983	7.41E-05	2.3	higher
32.93346	854.6843	TG 51:6|TG 15:2_18:2_18:2	139	1.16E-02	3.5	higher
36.80501	880.8327	TG 52:0|TG 16:0_18:0_18:0	908	7.32E-05	-2.8	lower
37.66025	872.7693	TG 52:4|TG 16:0_18:2_18:2	16	7.94E-03	2.1	higher
34.79023	877.7247	TG 52:4|TG 16:1_18:1_18:2	147	5.31E-05	-2.2	lower
34.27761	870.7531	TG 52:5|TG 16:0_18:2_18:3	24506	5.77E-08	2.4	higher
33.82025	868.7376	TG 52:6|TG 16:1_18:2_18:3	18373	2.66E-05	3.5	higher
33.33499	866.7216	TG 52:7|TG 16:1_18:2_18:4	1869	1.12E-03	4.3	higher
32.87514	864.7080	TG 52:8|TG 18:2_16:3_18:3	323	2.47E-04	4.5	higher
35.09014	886.7850	TG 53:4|TG 17:1_18:1_18:2	5077	8.25E-05	2.6	higher
34.56562	884.7698	TG 53:5|TG 17:1_18:2_18:2	1299	9.70E-04	3.1	higher
34.04579	882.7535	TG 53:6|TG 17:1_18:2_18:3	403	2.74E-03	3.0	higher
37.31323	908.8638	TG 54:0|TG 16:0_18:0_20:0	607	1.47E-05	-2.6	lower
37.04959	902.8176	TG 54:3|TG 18:0_18:1_18:2	19	1.00E-03	3.5	higher
33.62408	898.7857	TG 54:5|TG 18:1_18:2_18:2	98	3.39E-05	4.3	higher
34.36320	896.7689	TG 54:6|TG 18:1_18:2_18:3	6305	5.63E-04	2.6	higher
33.82173	899.7065	TG 54:7|TG 18:2_18:2_18:3	340	8.30E-05	2.8	higher
33.59570	892.7386	TG 54:8|TG 16:1_18:2_20:5	2405	9.94E-04	3.5	higher
33.33279	897.6937	TG 54:8|TG 18:1_18:3_18:4	172	4.66E-03	2.3	higher
33.13058	890.7228	TG 54:9|TG 16:1_16:2_22:6	349	1.22E-03	5.1	higher
37.08151	920.8655	TG 55:1|TG 16:0_21:0_18:1	250	2.57E-05	-2.9	lower
35.65592	914.8173	TG 55:4|TG 18:1_19:1_18:2	1471	5.92E-04	2.0	higher
37.80176	936.8950	TG 56:0|TG 16:0_18:0_22:0	161	8.80E-05	-2.5	lower
33.21978	916.7395	TG 56:10|TG 16:1_18:3_22:6	516	1.35E-03	3.5	higher
35.82156	932.8624	TG 56:2|TG 18:0_18:1_20:1	13687	8.88E-04	2.1	higher
34.82458	928.8307	TG 56:4|TG 20:0_16:2_20:2	8886	5.42E-05	2.7	higher
34.29202	925.7234	TG 56:8|TG 16:0_18:2_22:6	638	3.06E-04	-2.4	lower
33.75594	923.7072	TG 56:9|TG 16:1_18:2_22:6	198	8.16E-04	2.0	higher
36.83854	962.9116	TG 58:1|TG 18:0_22:0_18:1	846	3.17E-05	-2.1	lower
34.22786	944.7708	TG 58:10|TG 16:0_20:4_22:6	992	4.06E-04	-2.3	lower
38.87471	1071.004	TG 66:3|TG 16:0_32:1_18:2	72	2.04E-02	2.4	higher
39.14807	1085.020	TG 67:3|TG 16:1_18:1_33:1	38	2.63E-02	2.4	higher
39.40742	1099.034	TG 68:3|TG 16:0_34:1_18:2	249	2.79E-02	2.4	higher
38.92550	1097.020	TG 68:4|TG 18:1_32:1_18:2	257	7.31E-04	3.4	higher
39.19143	1111.035	TG 69:4|TG 18:1_33:1_18:2	48	4.60E-04	3.4	higher
39.45956	1125.050	TG 70:4|TG 18:1_34:1_18:2	95	1.03E-03	3.0	higher
39.01154	1123.0360	TG 70:5|TG 34:1_18:2_18:2	121	8.26E-05	2.7	higher

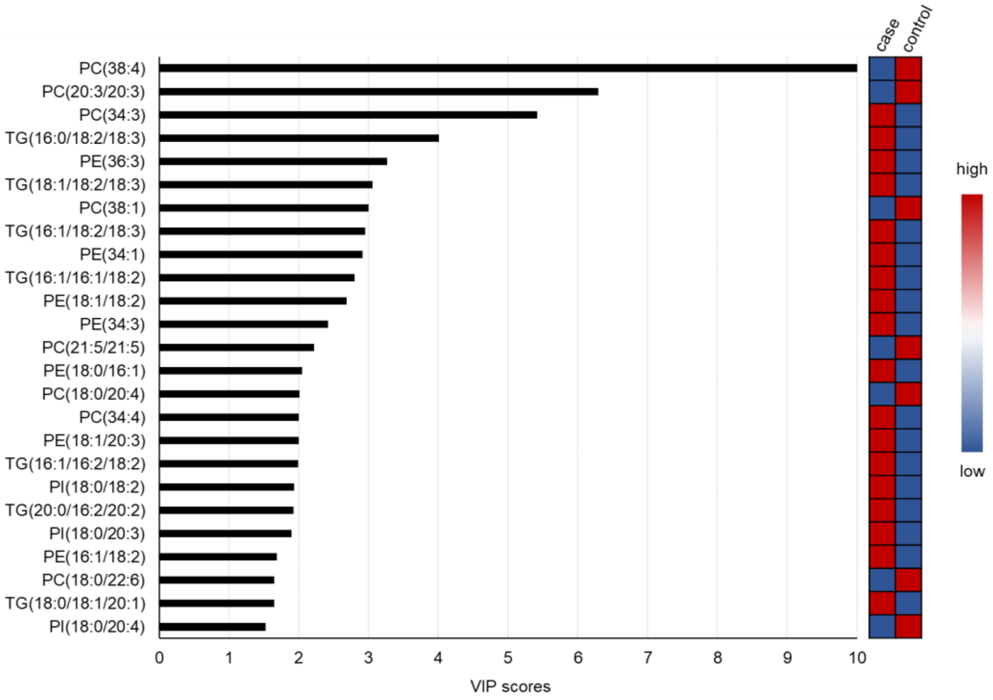

Figure S6. PLS-DA analysis of lipid species identified by LC-MS/MS: VIP score plot displays the top 25 most important lipids identified by PLS-DA. Colored boxes on the right indicate relative concentration of corresponding lipids for control and case samples.

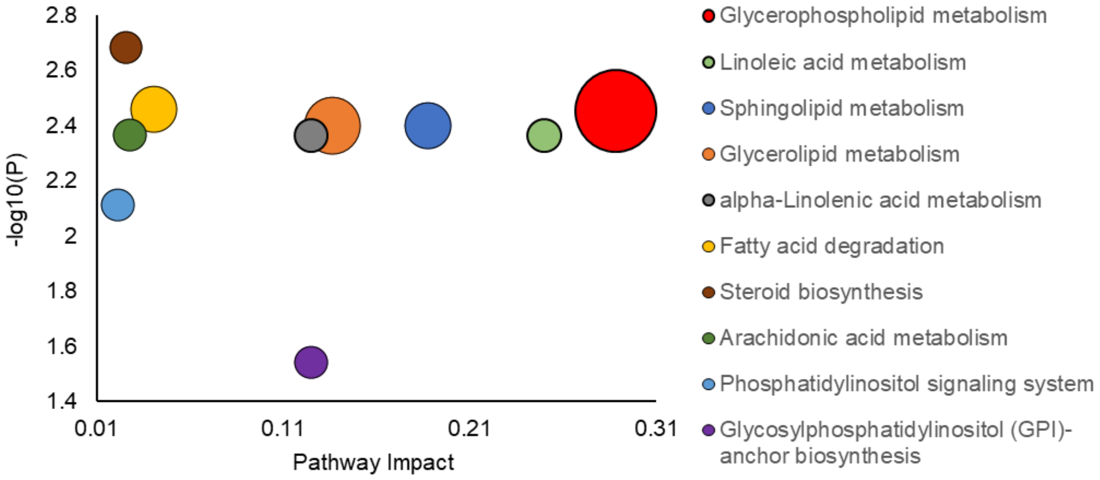

Figure S7. Metabolome view displays all significant pathways according to the p-values from the pathway enrichment analysis and pathway impact values (the sum of the importance measures of the matched metabolites normalized by the sum of the importance measures of all metabolites in each pathway) from the pathway topology analysis. The node size corresponds to the number of matched metabolites in each pathway and the node color corresponds to the pathway type.

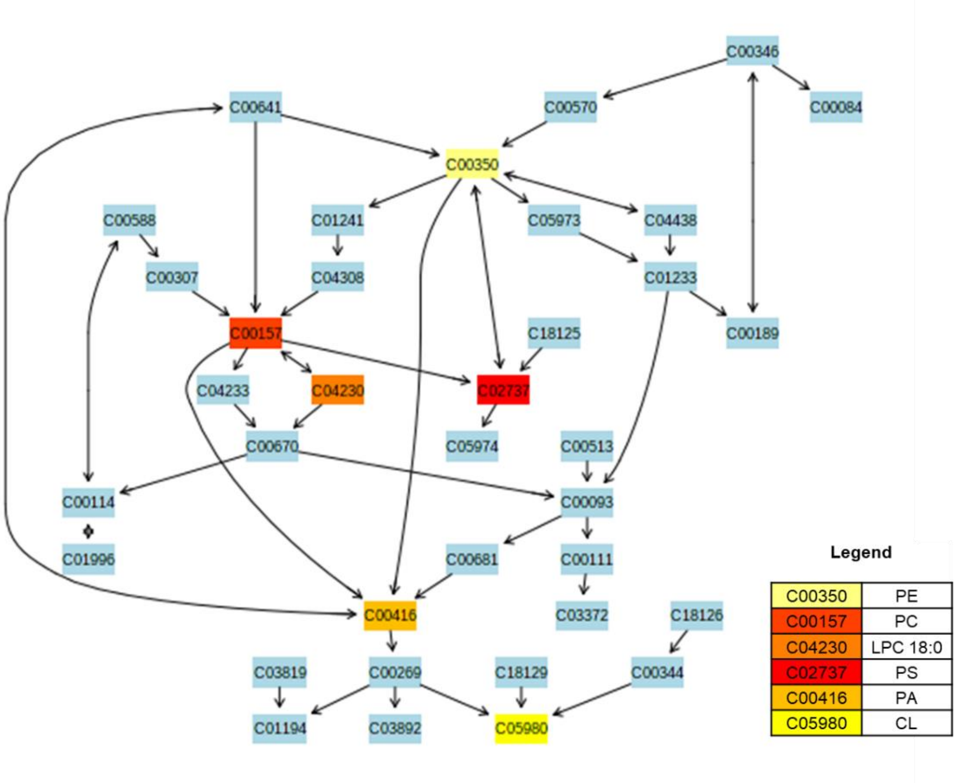

Figure S8. Glycerophospholipid metabolism. Boxes represent lipids with label name corresponding to KEGG metabolite ID. Blue boxes correspond to metabolites that are not in the dataset while other colors (varying from yellow to red) correspond to the metabolites that are in the dataset with different levels of significance.

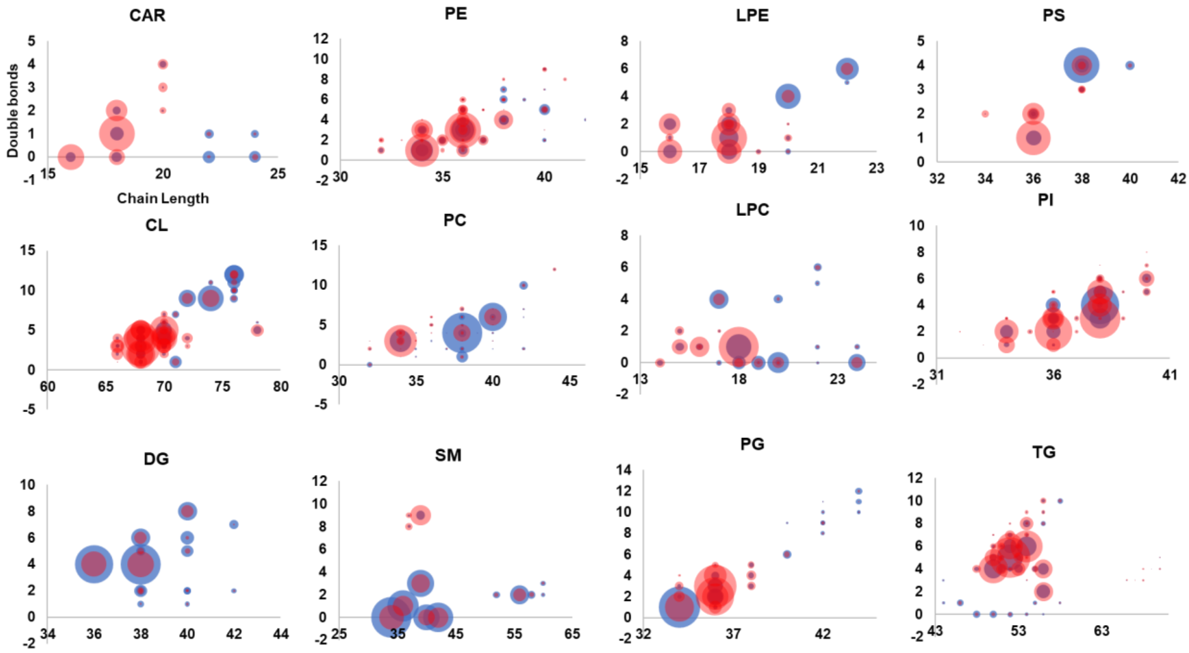

Figure S9. Chain length – double bond abundance for the main lipid classes. The dot sizes indicate the lipid abundance, and the dot colors correspond to the control (blue) or case (red) samples.

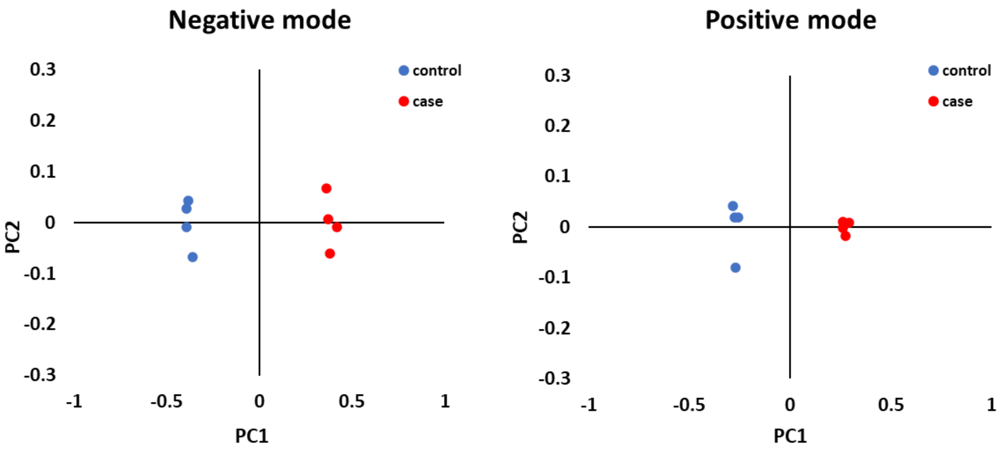

Figure S10. Two dimensional PCA scores of lipid species identified in the cross-sections of the control (blue) and case (red) samples by MALDI in negative and positive modes.

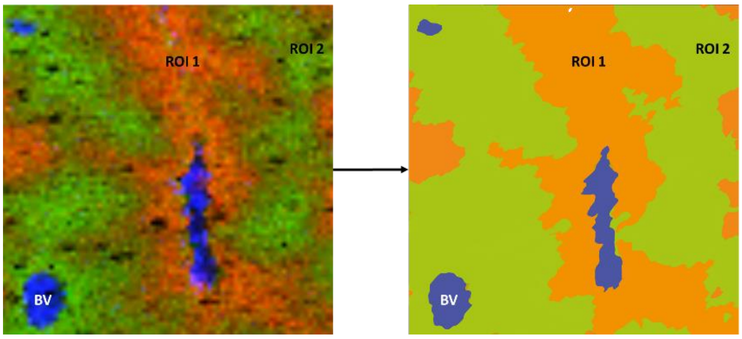

Figure S11. Principle of image segmentation: image subdivision in three different regions of interest (ROIs): ROI1 in orange, ROI2 in green and BV (= blood vessel) in blue.

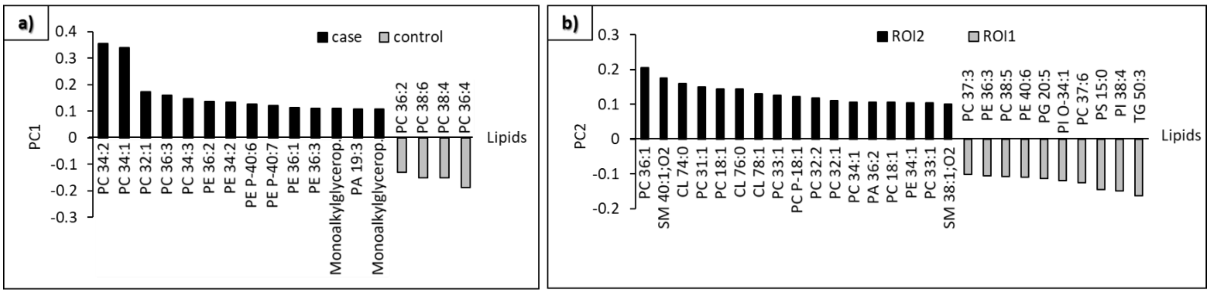

Figure S12. Statistical analysis on lipids identified by MALDI in positive mode. (a) PCA loading plot of the lipids contributing most to the variation (PC1 ≥ 0.1 or ≤ -0.1) between case and control mouse liver. Lipids with a positive PC1 value have increased levels after PFOA exposure (black bars) while lipids with a negative PC1 value have decreased levels after exposure (grey bars). (b) PCA loading plot of the lipids contributing most to variation (PC2 ≥ 0.1 or ≤ -0.1) between ROI1 and ROI2 isolated in the images of the case samples. Lipids with a positive PC2 value have higher levels in ROI2 (black bars) while lipids with a negative PC2 value have higher levels in ROI1 (grey bars).

Table S5. Comparative study between MALDI and LC-MS/MS results (lipid selection from MALDI results in negative mode with PC1 values > 0.05 or < -0.05): identification and correlation. Lipid species which are differently or not identified by LC-MS/MS are highlighted in grey.

MALDI				LC-MS/MS				
m/z	Compound ID	PC1	Group	RT (min)	*m/z*	Compound ID	Correlation factor	Group
885.5498	PI 38:4	-0.36	control	22.53	885.5464	PI 38:4|PI 18:0_20:4	-0.28	control
766.5395	PE 38:4	-0.22	control	26.98	766.5387	PE 38:4|PE 18:0_20:4	-0.93	control
790.5394	PE 40:6	-0.12	control	24.56	790.5381	PE 40:6|PE 18:0_22:6	-0.55	control
723.4973	PA 38:4	-0.11	control	23.29	723.4968	w/o MS2:PA 10:0_28:4	-0.68	control
794.5704	PE 40:4	-0.10	control	28.25	794.5687	PE 40:4|PE 20:0_20:4	-0.76	control
834.5282	PS 40:6	-0.10	control	34.17	834.5278	PS 40:6|PS 18:0_22:6	-0.45	control
762.508	PE 38:6	-0.10	control	24.34	762.5067	PE 38:6|PE 18:2_20:4	-0.75	control
810.5289	PS 38:4	-0.10	control	32.35	810.5286	PS 38:4|PS 18:0_20:4	-0.91	control
857.5186	PI 36:4	-0.09	control	21.06	857.5167	PI 36:4	-0.41	control
770.5709	PE 38:2	-0.08	control	28.44	770.5699	PE 38:2|PE 20:0_18:2	-0.39	control
419.2568	CPA 18:0	-0.08	control					
695.4659	PA 36:4	-0.08	control	22.60	419.313	w/o MS2:AAHFA 18:3/8:0;O	-0.92	control
719.4657	PA 38:6	-0.07	control	28.62	719.4652	w/o MS2:PA 10:0_28:6	0.86	case
508.3407	PE 20:0	-0.07	control	13.24	508.3405	w/o MS2:PE O-8:0_12:0	-0.64	control
437.2674	PA 18:0	-0.07	control	15.54	437.2659	w/o MS2:NAGlySer 18:3;O	-0.92	control
721.4814	PA 38:5	-0.07	control					
599.3203	PI 18:0	-0.07	control	10.74	599.3197	LPI 18:0	0.48	case
747.5177	PG 34:1	-0.06	control	23.28	747.5181	PG 34:1|PG 18:0_16:1	-0.66	control
818.5702	PE 42:6	-0.06	control	27.89	818.5698	PE 42:6|PE 20:0_22:6	-0.89	control
771.6382	SM 39:1	-0.06	control	28.97	771.6376	w/o MS2:PE-Cer 20:1;2O/22:0	-0.91	control
745.4812	PA 40:7	-0.06	control					
747.5177	PG 34:1	-0.06	control	23.28	747.5181	PG 34:1|PG 18:0_16:1	-0.66	control
391.2257	CPA 16:0	-0.06	control					
792.5546	PE 40:5	-0.06	control	27.44	792.5504	PE 40:5|PE 18:0_22:5	-0.91	control
687.5443	SM 33:1	-0.06	control					
749.5123	PA 40:5	-0.05	control					
718.5393	PC 31:0	-0.05	control					
771.518	PG 36:3	0.05	case	22.30	771.5153	PG 36:3|PG 18:1_18:2	0.75	case
745.5023	PG 34:2	0.05	case	20.84	745.501	PG 34:2|PG 16:1_18:1	0.81	case
786.5289	PS 36:2	0.05	case	29.18	786.5285	PS 36:2|PS 18:0_18:2	0.86	case
736.4921	PE 36:5	0.05	case	24.32	736.4905	PE 36:5|PE 16:1_20:4	0.70	case
863.5639	PI 36:1	0.06	case	24.46	863.5643	PI 36:1|PI 18:0_18:1	0.86	case
731.4856	PG 33:2	0.06	case	24.62	731.4849	w/o MS2:PG 7:0_26:2	0.68	case
571.2888	PI 16:0	0.06	case	6.95	571.2882	LPI 16:0	0.45	case
730.5026	PS 33:2	0.06	case	23.64	730.5021	w/o MS2:PE O-12:0_22:3;2O	0.75	case
688.4918	PE 32:1	0.06	case	25.33	688.4914	PE 32:1|PE 14:0_18:1	0.54	case
645.4499	PA 32:1	0.07	case					
669.4499	PA 34:3	0.07	case	29.24	669.4497	w/o MS2:PA 8:0_26:3	0.86	case
756.5182	PS 35:2	0.07	case	20.94	756.5178	PE 36:3;O|PE 18:1_18:2;O	0.89	case
758.5339	PS 35:1	0.07	case	19.68	758.5328	w/o MS2:PE O-14:0_22:3;2O	0.81	case
597.3044	PI 18:1	0.07	case	6.96	597.3032	w/o MS2:LPI 18:1	0.68	case
738.5081	PE 36:4	0.07	case	24.59	738.5082	PE 36:4|PE 18:2_18:2	0.93	case
476.2783	PE 18:2	0.08	case	8.19	476.2783	LPE 18:2	0.32	case
699.4976	PA 36:2	0.09	case	23.41	699.4963	PEtOH 34:2	0.72	case
478.2939	PE 18:1	0.10	case	10.55	478.2932	LPE 18:1	0.69	case
883.5342	PI 38:5	0.11	case	21.58	883.5323	PI 38:5|PI 18:1_20:4	0.83	case
712.4923	PE 34:3	0.11	case	24.21	712.4919	PE 34:3|PE 16:1_18:2	0.84	case
768.5549	PE 38:3	0.11	case	27.47	768.5524	PE 38:3|PE 18:0_20:3	0.76	case
835.5344	PI 34:1	0.11	case	23.02	835.5337	PI 34:1|PI 16:0_18:1	0.57	case
697.4817	PA 36:3	0.13	case					
452.2785	PE 16:0	0.13	case	10.69	452.2777	LPE 16:0	0.80	case
673.4818	PA 34:1	0.13	case	36.11	673.4818	w/o MS2:PA 8:0_26:1	0.82	case
859.5342	PI 36:3	0.14	case	21.80	859.5331	PI 36:3|PI 18:1_18:2	0.84	case
833.5186	PI 34:2	0.14	case	21.60	833.5173	PI 34:2	0.74	case
861.55	PI 36:2	0.16	case	21.07	861.5516	PI 36:2	0.91	case
716.5241	PE 34:1	0.16	case	26.78	716.5226	PE 34:1|PE 18:0_16:1	0.85	case
671.466	PA 34:2	0.17	case	36.12	671.4663	PA 34:2|PA 16:1_18:1	0.82	case
887.5661	PI 38:3	0.17	case	23.71	887.5626	PI 38:3|PI 18:0_20:3	0.81	case
744.5559	PE 36:1	0.19	case	28.11	744.553	PE 36:1|PE 18:0_18:1	0.93	case
740.5242	PE 36:3	0.22	case	35.60	740.522	PE 36:3|PE 18:1_18:2	0.91	case
742.5396	PE 36:2	0.24	case	35.60	742.5378	PE 36:2|PE 18:0_18:2	0.93	case
714.5081	PE 34:2	0.30	case	35.58	714.5067	PE 34:2|PE 16:0_18:2	0.81	case

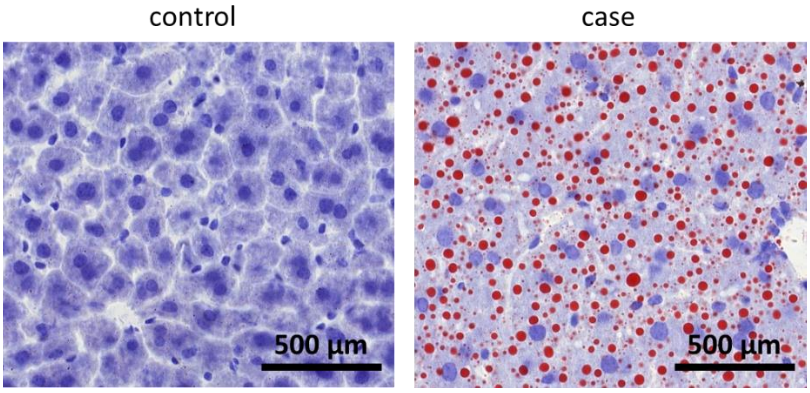

Figure S13. Light microscopy images of control and case samples labelled with Oil-Red-O enabling the visualization of lipid droplets in the tissue.

REFERENCES

(1) Kenwood, B. M.; Merrill, A. H. Lipidomics. *Encycl. Cell Biol.* **2016**, *1*, 147–159. https://doi.org/10.1016/B978-0-12-394447-4.10024-0.
